# Updating Urinary Microbiome Analyses to Enhance Biologic Interpretation

**DOI:** 10.3389/fcimb.2022.789439

**Published:** 2022-07-08

**Authors:** Nazema Y. Siddiqui, Li Ma, Linda Brubaker, Jialiang Mao, Carter Hoffman, Erin M. Dahl, Zhuoqun Wang, Lisa Karstens

**Affiliations:** ^1^ Division of Urogynecology & Reconstructive Pelvic Surgery, Division of Reproductive Sciences, Department of Obstetrics & Gynecology, Duke University Medical Center, Durham, NC, United States; ^2^ Department of Statistical Science, Duke University, Durham, NC, United States; ^3^ Department of Biostatistics and Bioinformatics, Duke University School of Medicine, Durham, NC, United States; ^4^ Division of Female Pelvic Medicine and Reconstructive Surgery, Department of Obstetrics, Gynecology and Reproductive Sciences, University of California, San Diego, San Diego, CA, United States; ^5^ Division of Bioinformatics and Computational Biomedicine, Department of Medical Informatics and Clinical Epidemiology, Oregon Health & Science University, Portland, OR, United States; ^6^ Division of Urogynecology, Department of Obstetrics and Gynecology, Oregon Health & Science University, Portland, OR, United States

**Keywords:** urinary microbiome, urobiome, bioinformatic analysis, mixed urinary incontinence, bladder dysfunction, lactobacilli, microbiota

## Abstract

**Objective:**

An approach for assessing the urinary microbiome is 16S rRNA gene sequencing, where analysis methods are rapidly evolving. This re-analysis of an existing dataset aimed to determine whether updated bioinformatic and statistical techniques affect clinical inferences.

**Methods:**

A prior study compared the urinary microbiome in 123 women with mixed urinary incontinence (MUI) and 84 controls. We obtained unprocessed sequencing data from multiple variable regions, processed operational taxonomic unit (OTU) tables from the original analysis, and de-identified clinical data. We re-processed sequencing data with DADA2 to generate amplicon sequence variant (ASV) tables. Taxa from ASV tables were compared to the original OTU tables; taxa from different variable regions after updated processing were also compared. Bayesian graphical compositional regression (BGCR) was used to test for associations between microbial compositions and clinical phenotypes (e.g., MUI versus control) while adjusting for clinical covariates. Several techniques were used to cluster samples into microbial communities. Multivariable regression was used to test for associations between microbial communities and MUI, again while adjusting for potentially confounding variables.

**Results:**

Of taxa identified through updated bioinformatic processing, only 40% were identified originally, though taxa identified through both methods represented >99% of the sequencing data in terms of relative abundance. Different 16S rRNA gene regions resulted in different recovered taxa. With BGCR analysis, there was a low (33.7%) probability of an association between overall microbial compositions and clinical phenotype. However, when microbial data are clustered into bacterial communities, we confirmed that bacterial communities are associated with MUI. Contrary to the originally published analysis, we did not identify different associations by age group, which may be due to the incorporation of different covariates in statistical models.

**Conclusions:**

Updated bioinformatic processing techniques recover different taxa compared to earlier techniques, though most of these differences exist in low abundance taxa that occupy a small proportion of the overall microbiome. While overall microbial compositions are not associated with MUI, we confirmed associations between certain communities of bacteria and MUI. Incorporation of several covariates that are associated with the urinary microbiome improved inferences when assessing for associations between bacterial communities and MUI in multivariable models.

## Introduction:

The urinary microbiome is being investigated in multiple bladder conditions. There are now several reports demonstrating differences in urinary microbiota in women with recurrent urinary tract infection (UTI) (Burnett et al., 2021; Vaughan et al., 2021), urgency urinary incontinence (Pearce et al., 2014; Karstens et al., 2016), and mixed urinary incontinence (Komesu et al., 2018) when compared to matched controls without these symptoms. In most of these studies, 16S rRNA amplicon sequencing has been employed as a culture-independent method of detecting urinary bacteria. When using sequencing to detect bacteria, DNA is extracted from a biological sample, polymerase chain reaction (PCR) is used to amplify and sequence portions of the 16S rRNA gene, and bioinformatic tools are used to match the recovered sequences with those existing in a reference database. Results are reported as taxonomic groupings (i.e., taxa). These steps allow investigators to identify the bacteria contained in a sample. Next, the recovered taxa can be compared between participant cohorts using statistical analyses to discern if there are differences between phenotypic groups.

The bioinformatic steps outlined above depend on multiple computational components, which have been rapidly evolving. Many prior analyses were performed with reference databases that have not been recently updated, such as Greengenes^1^. Furthermore, the Greengenes database does not have substantial representation of urinary bacteria and thus may not be the optimal reference database for identification of microbiota within a urine sample (Hoffman et al., 2021). Regardless of the reference database that is selected, bioinformatic workflows rely on algorithms that group raw sequencing data based on similarities (Knight et al., 2018). These algorithms are rapidly evolving, and when updated or refined, could potentially alter bacterial identification results (Kopylova et al., 2016; Callahan et al., 2017; Edgar, 2017; Nearing et al., 2018; Caruso et al., 2019). Previously, researchers would group raw sequences into operational taxonomic units (OTUs) based on similarity, then compared these OTUs against reference databases to identify the bacterial taxa (Westcott and Schloss, 2015). Many now advocate for grouping raw sequences using amplicon sequence variant (ASV)-based methods, where sequences are grouped based on their error-corrected exact sequences (Callahan et al., 2017). In ASV-based methods, the error score assessing the confidence of sequencing results at each nucleotide is incorporated such that algorithms can better detect true biologic sequences versus those generated by sequencing error. Furthermore, ASV-based methods have the ability to identify bacterial taxa at finer resolution (e.g., genus and species levels where previously identifications were at higher taxonomic levels such as the family level). Studies that were performed prior to these updates in bioinformatic workflows may benefit from re-analysis.

Separate from bioinformatic components of analyses, the statistical methods used to analyze microbial data are also evolving. Prior studies have used methods such as Dirichlet Multinomial Mixture (DMM) modeling (Holmes et al., 2012), which adopt simplistic distributional assumptions on the microbiome compositions, and linear discriminant analysis effect size (LeFSE)^2^, which is a nonparametric cross-sample test that utilizes linear discriminant analysis (LDA) to construct test statistics assisted by classical univariate tests for feature (e.g., organism, clade, or OTU) selection. Neither approach adequately accounts for all of the key characteristics of microbiome data such as their compositional constraints, complex cross-sample heterogeneity, and sparse counts of certain taxa. Thus, high dimensional microbial datasets that are used in these types of compositional or community-based analyses fail to meet the underlying assumptions that are needed for the statistical techniques. Rather, more recently developed tree-based models (Wang and Zhao, 2017; Mao et al., 2020), community-based analyses (Layeghifard et al., 2017), or other models that more truthfully account for the distributional characteristics may be needed, especially in view of the limited sample sizes in most studies. These modeling techniques are currently under further development and may be able to better detect the true signal within a dataset.

For datasets with robust findings, updated analytic techniques should not substantially alter major findings. However, urinary microbiome results could be especially prone to bias or skew from different analytic techniques, since small differences are magnified in low biomass environments (Caruso et al., 2019). We hypothesized that updated analyses would enhance precision and allow for more clarity with biologic inferences and thus we used updated techniques to re-analyze raw sequencing data generated in a prior study (Komesu et al., 2018). Our primary objective was to determine whether taxonomic identifications substantially differ with an updated bioinformatic pipeline. Secondary objectives were to compare taxonomic identifications based on the 16S rRNA gene variable region used, to assess whether tree-based modeling strategies enhance our ability to differentiate microbial community profiles between women with mixed urinary incontinence and controls, and re-assess relationships between bacterial communities and mixed urinary incontinence with updated information.

## Methods

**Figure 1:**
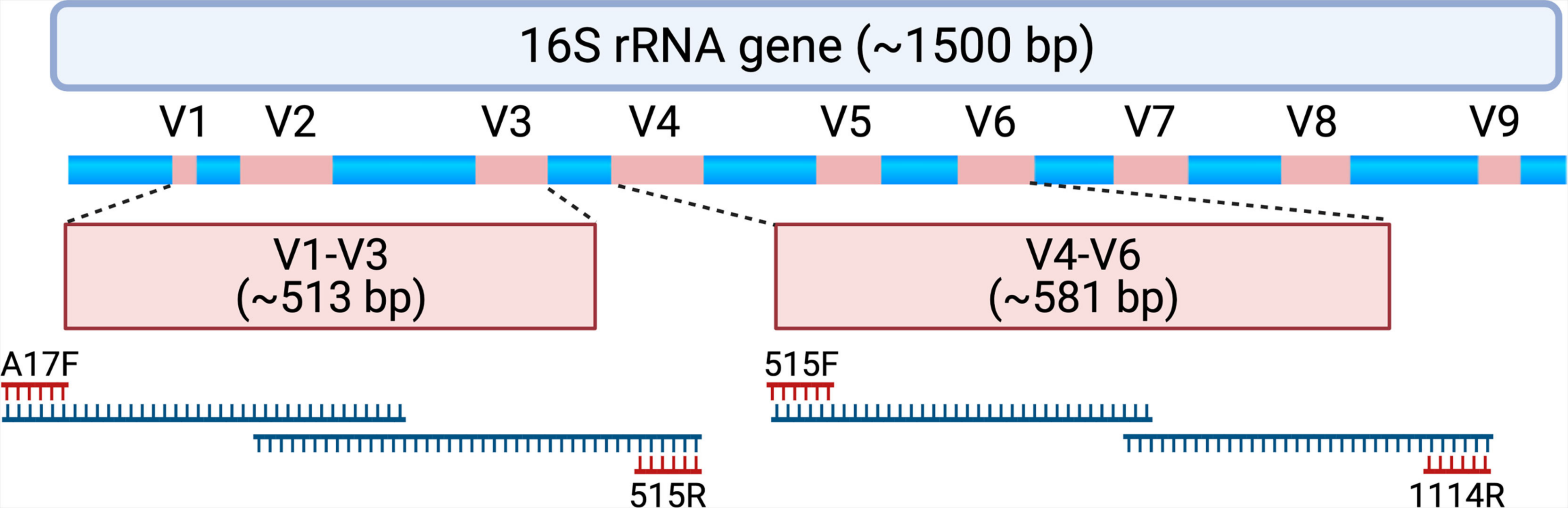
Schematic depicting the 16S rRNA gene and approximate locations of variable regions (V1-V9) that can be selected for amplicon-based sequencing. Samples assessed for this study underwent polymerase chain reaction (PCR) to amplify the V1-V3 region using PCR primers A17F and 515R. When forward and reverse reads are merged, the entire V1-V3 region spans 513 ± 22 base pairs with approximately 87 base pairs of overlapping sequence. Samples also underwent PCR to amplify the V4-V6 region using PCR primers 515F and 1114R. When forward and reverse reads are merged, the entire V4-V6 region spans 581 ± 2 base pairs with approximately 19 base pairs of overlapping sequence. For this study, forward and reverse reads were generated on an Illumina MiSeq platform, which creates sequencing reads of approximately 300 base pairs in length. The initial and final portions of each sequencing read tend to contain lower quality sequence (i.e., lower confidence scores with nucleotide assignment) that could be adjusted or truncated in a DADA2 processing pipeline. As such, paired end reads without a substantial amount of overlapping sequencing may not be able to be merged. Created with BioRender.com.

After Duke University Institutional Review Board approval (Pro #00102155), we conducted a re-analysis of sequencing data generated from the Human Microbiome Study in the Effects of Surgical Treatment Enhanced with Exercise for Mixed Urinary Incontinence HMS-ESTEEM Study (Komesu et al., 2018). The HMS-ESTEEM study was a supplemental translational study embedded within the ESTEEM randomized trial (Sung et al., 2019) conducted by 8 clinical sites within the Pelvic Floor Disorders Network (PFDN)^3^. Briefly, this was a cross-sectional analysis of microbiome data obtained from women with mixed urinary incontinence (MUI) and age-matched controls. The strict inclusion and exclusion criteria for participants (207 women, 123 with MUI and 84 age-matched controls) are detailed in prior publications (Sung et al., 2016; Komesu et al., 2017). Women completed validated questionnaires to assess urinary symptom burden and to confirm appropriate categorization into MUI and control groups. Additional questionnaires were administered to gather data about hormonal therapies, sexual activity, recent infections, and the presence of any vaginal medications. Urine samples were obtained *via* transurethral catheterization and stored in a DNA protectant (Assay Assure™, Sierra Molecular Corporation, Incline Village, NV, USA). Samples were transferred with cold packs *via* overnight shipping to a central laboratory where they were immediately processed, and DNA was extracted. DNA was stored at -80°C prior to sequencing until all samples were collected. DNA was then thawed, subjected to polymerase chain reaction (PCR) amplification and 16S rRNA gene sequencing. For each sample, two separate 16S rRNA gene regions (i.e., the V1-V3 and V4-V6 hypervariable regions) were sequenced (**Figure 1**). Laboratory methods, primers, and details regarding a multi-step PCR (total 38 cycles) are described in detail in a methodology paper associated with the original study (Komesu et al., 2017).

### Bioinformatics

**Figure 2:**
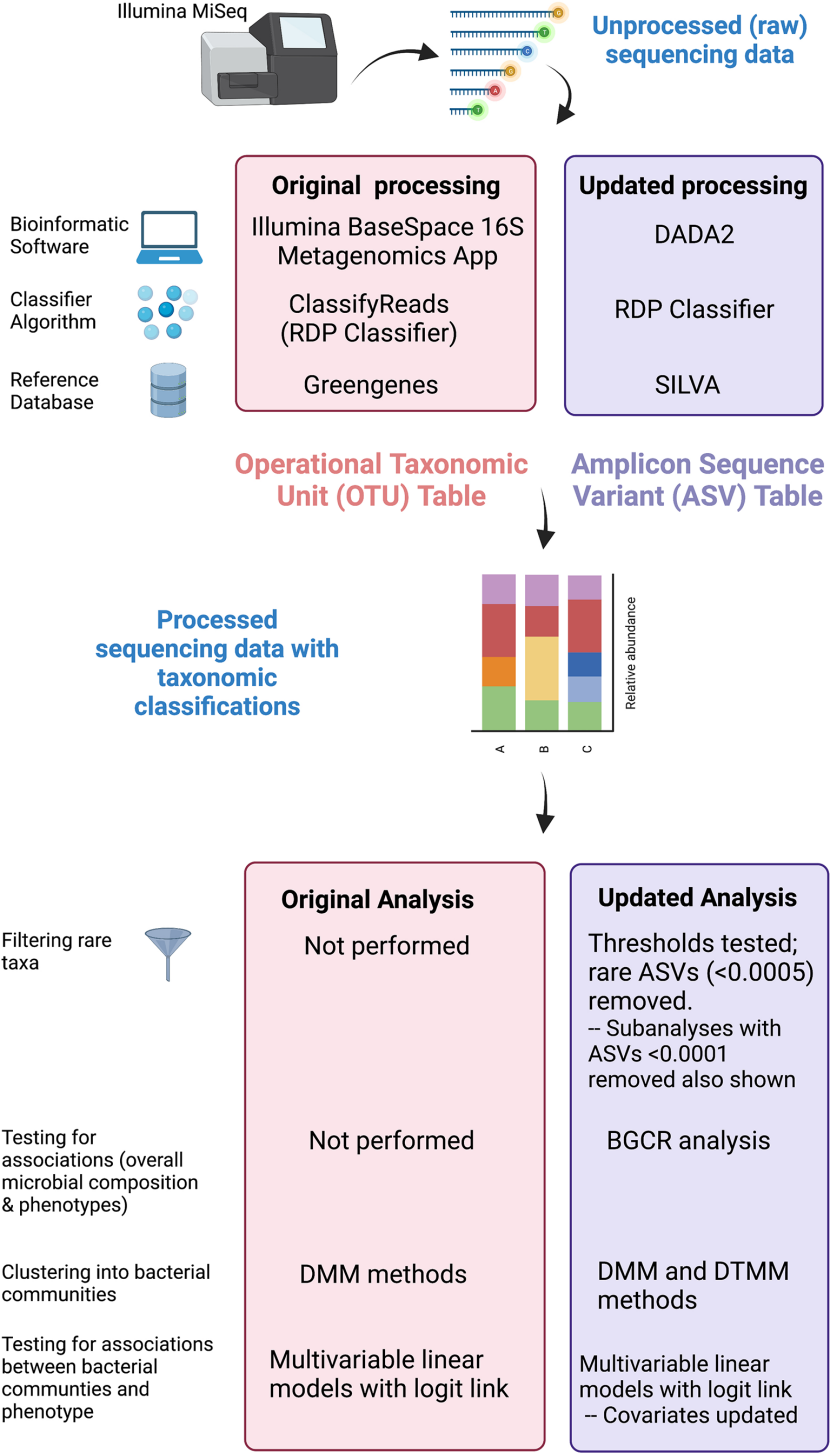
Comparison of original and updated methods. Unprocessed sequencing data generated from an Illumina MiSeq platform were obtained. The figure illustrates differences in the bioinformatic software, classifying algorithm, and reference databases between original and updated processing. The output from original processing was an OTU table. The output from updated processing was an ASV table, which is similar to an OTU table but also adjusts for sequencing errors. Both tabular outputs can be incorporated into Phyloseq objects in R to visualize data as stacked bar plots. Original and updated statistical analysis methods including filtering, clustering, and methods used to test for associations are also listed. Created with BioRender.com.

We obtained unprocessed sequencing files from the Sequence Read Archive^4^ Bioproject #703967 previously generated OTU tables, and associated clinical data from the PFDN data coordinating center. First, we repeated sequence processing using updated techniques. Differences between original and updated processing are illustrated in **Figure 2**. In the original analysis, sequencing data were processed using the Illumina BaseSpace 16S Metagenomics App version 1.0.1. This software classifies raw sequencing data using ClassifyReads, a high-performance implementation of the Ribosomal Database Project (RDP) classifier (Wang et al., 2007), and compares classified sequence reads against the Greengenes reference database to identify bacteria. The output is an OTU table, which is a designation of relative proportions of different taxonomic groups that each sample contains. In the updated analysis, raw sequences files were processed using the learnErrors and derepFastq functions in DADA2 (Callahan et al., 2016)(v 1.14.0) with default parameters, then mapped to the SILVA reference database (Quast et al., 2013)(v 132) with the RDP classifier implemented in the assignTaxonomy function in DADA2. The end result is an ASV table, which is similar to an OTU table but takes into account sequencing error rather than sequence similarity when grouping sequences together. Data were further processed and visualized in R using phyloseq (McMurdie and Holmes, 2013) (v. 1.26.1) and microshades (Dahl et al., 2021) (v. 0.0.0.9).

### Comparisons of Recovered Taxa in Original Versus Updated Analysis

Using phyloseq and microshades in R, plots were generated to illustrate the recovered taxa identified in the original analysis (i.e., OTU table) and updated analysis (i.e., ASV table). Notably, these comparisons were performed on unfiltered data. Relative abundances of recovered taxa per sample were calculated for original and updated analyses, and directly compared to assess for differences in recovered taxa based on bioinformatic processing.

### Comparisons of Recovered Taxa by Sequencing Amplicons

When performing 16S rRNA gene sequencing, generally one or more variable regions (commonly V1-3 or V4-6) of the 16S rRNA gene are amplified and sequenced; the variable regions chosen are referred to as amplicons. Different amplicons contain distinct regions of DNA, vary in length, and may have different representation in reference databases. As such, one amplicon may identify a specific bacterium at higher resolutions or in a more specific manner than another amplicon targeting a different variable region. Each DNA sample in this study was subjected to sequencing using two different amplicons, one targeting the V1-V3 variable region and the other targeting the V4-V6 variable region. We compared taxa identified from the same DNA sample using both amplicons to understand which taxa might be differentially identified in urine based on the amplicon chosen for sequencing. Data were visualized in R using phyloseq and microshades. Plots were generated to illustrate the recovered taxa in samples with paired V1-V3 and V4-V6 amplicon data. Relative abundances of recovered taxa per sample were calculated for each amplicon and were compared between amplicons.

### Rare ASVs: Filtering Thresholds

ASV tables generated from the updated bioinformatic analysis were incorporated into downstream statistical analyses, including clustering to assess for latent community structure. Notably, in the original study, DMM clustering was performed on unfiltered data. However, given the low biomass sample type and potential for contaminants to influence results, we chose to filter data prior to clustering. Data were filtered in R based on the relative abundance of read counts with taxa below a specified threshold removed from the dataset. We used a hybrid of the “Rule of Thumb” and “Statistical Threshold” methods that have been previously reported (Cao et al., 2020). After initial review, the microbial data contained within this dataset were mainly dominated by a few ASVs with many other ASVs having low relative abundances. Thus, we assessed a range of filtering thresholds from 0.05 - 0.0001 and the subsequent effects on downstream clustering for the entire dataset (i.e., prior to assigning MUI versus control labels). Coarse filtering thresholds of 0.05 – 0.001 resulted in removal of some low abundance taxa (including *Escherichia/Shigella*) that have been demonstrated in other urinary microbiome studies and are inferred as being true representatives of the urinary microbiome. Furthermore, when using coarse thresholds, we observed non-informative clusters that did not appear biologically distinct. We selected a more conservative filtering threshold of 0.0005, though in several places we illustrate how a less restrictive threshold of 0.0001, which more closely approximates unfiltered data, might have influenced downstream results.

### Testing for Associations Between Individual Taxa and Clinical Phenotypes

In the original study by Komesu *et al. (* Komesu et al., 2018), the primary aim focused on differences in *Lactobacillus* predominance between clinical phenotypes (i.e., MUI and control). However, there were no methods employed to determine whether other specific taxa are associated with MUI vs. control. Bayesian graphical compositional regression (BGCR) is a technique that allows us to test for associations of individual taxa (including rare taxa) with outcomes (Mao et al., 2020). BGCR models the distribution of microbiome data while incorporating phylogenetic relationships and adjusts for other variables that could potentially confound associations with outcomes. BGCR inherently controls for multiple testing and returns a posterior joint alternative probability (PJAP) with a larger PJAP (closer to 1) indicating evidence of differences in taxa between groups. We tested for differences in taxa between MUI and control participants using this technique. BGCR (Mao et al., 2020) was performed in R using BGCR v0.1.0. For BGCR analyses we included the same covariates that we later included in models assessing community structure and phenotypes. These were: age, smoking status, ethnicity, body mass index (BMI), composite menopausal/hormonal status, vaginal pH, history of recurrent UTI, and number days from the most recent catheterization. The strategy behind variable selection is further discussed below and in **Supplemental Table 1**.

### Clustering Into Bacterial Communities

Typically, a microenvironment (e.g., urinary bladder) will contain several taxa that are considered together as a bacterial community. While some conditions might have unique bacterial taxa associated a phenotype, in other conditions, there may be overarching differences in bacterial community structure that are associated with phenotypes. Thus, when trying to infer clinical implications from microbial datasets, samples are often clustered based on those that contain similar combinations of taxa, resulting in a substructure of several bacterial communities. Bacterial communities (rather than individual taxa) can then be assessed for associations with clinical phenotypes. In the original analysis, unfiltered taxa were clustered into bacterial communities using Dirichlet Multinomial Mixture (DMM) (Holmes et al., 2012), and these communities were subsequently tested for associations with MUI vs. control phenotypes. In DMM methods, investigators assign the number of clusters (corresponding to bacterial communities) that are desired. This is achieved by reviewing results with different numbers of clusters and selecting the final number of clusters that qualitatively seems to make sense. We repeated DMM clustering on ASV data from the updated bioinformatic analysis in R using DirichletMultinomial v 1.36.0. We chose the same number of clusters that were selected in the original publication. However, since selecting the number of clusters can introduce bias, we also evaluated another method of clustering that automatically adapts the number of clusters based on the complexity of the data. This is a nonparametric mixture model that utilizes the phylogenetic tree to enrich the modeling on cross-sample variability called Dirichlet tree multinomial mixture (DTMM) (Mao and Ma). We performed this clustering method in R using DTMM v0.1.0^5^. While DMM clustering is highly influenced by the “dominant”, or most abundant taxa in a sample, DTMM more effectively incorporates less abundant taxa. Whether obtained through DMM or DTMM methods, final clusters are considered bacterial communities that can be tested for associations with phenotypes.

### Testing for Associations Between Bacterial Communities and MUI Versus Control Phenotype

Similar to the original analysis, in this updated analysis we assessed for associations between bacterial communities and clinical phenotype (MUI vs. control) using multivariable generalized linear models with a logit link. While our primary analysis focused on bacterial communities generated from DMM methods, we also assessed models that incorporated bacterial communities generated through DTMM clustering methods to assess how clustering methods might influence results. Both original and updated analyses incorporated several covariates though some of these were chosen differently, as detailed in **Supplemental Table 1**. In both original and updated analyses, bacterial community types were included in models with the following covariates: age, ethnicity, BMI, and smoking status. However, in the original analysis age was strongly associated with bacterial communities. Thus, investigators performed *post hoc* sub-analyses in participants < 51 years and those with ages 51 and older, with the age of 51 chosen since it is the median age of menopause in the United States (Komesu et al., 2018). For the updated analysis, we elected to include menopausal status into our model, but also needed to appropriately manage hormone therapy that occurs with menopause. To do this, we created a composite variable that incorporated menopausal and hormonal status as one of three categorical options: 1) pre-menopausal; 2) post-menopausal with any estrogen hormone use (topical, vaginal, transdermal, oral); and 3) post-menopausal without hormone use. In addition, we included vaginal pH in updated models. Finally, we added two covariates into updated models because of their relevance to the microbiome - history of recurrent UTI, and number of days from the most recent catheterization (calculated based on last prior recorded catheterized urine sample or urodynamic assessment). These variables have been proposed as “desired” within recently published standards for urinary microbiome research (Brubaker et al., 2021). Both the original and updated analyses considered clinical site where samples were acquired, though site was managed differently in original versus updated models (see **Supplemental Table 1** & **Supplemental Figure 1**). Multivariable modeling was performed in R using stats v4.0.5.

## Results

Unprocessed sequencing data from 207 samples (123 MUI and 84 controls) that were sequenced using 300bp paired-end reads from V1-V3 and V4-V6 variable regions were reprocessed for this analysis. This resulted in taxonomic data in 173 samples for the V1-V3 region and 194 samples for the V4-V6 region. When attempting to merge forward and reverse reads from the V1-V3 region, there was substantial data loss such that reads from approximately 25% of the samples would have been removed from the dataset. Similarly, sequencing reads from the V4-V6 region, which provides a longer amplicon, were unable to be merged because of lack of enough overlapping sequence (see **Figure 1**). Given these constraints in data from both amplicons, we used forward reads only for subsequent analyses. It is unclear if the reads were merged or unmerged in the original analysis which used the Illumina BaseSpace 16S Metagenomics App for sequence processing. Median classified reads (i.e. recovered taxa) from the ASV table are summarized in **Table 1** and compared to those from the original analysis. For both amplicons in this dataset, we observed a median of 301 base pairs (bp) in sequencing read length. Given that we used forward reads only, we assessed the variable regions that would have been spanned with the stated primers and ~301 bp of sequencing (see **Figure 1**). As such, the V1-V3 forward read covers all of V1 and most of the V2 region. The V4-V6 forward read mainly comprises the V4 region, as it is not long enough to span V5 and V6 regions. For improved transparency and accuracy, in the remainder of this manuscript we will refer to the data as those arising from the V1-V2 regions and V4 regions, respectively.

**Table 1:** Sequencing data & Recovered taxa.

	Median (range) classified reads	# Phyla	# Classes	# Orders	# Families	# Genera
V1-V2^*^	24,862 (1,021 – 670,442)	29	63	143	220	545
V4^*^	29,105 (5,029 – 187,593)	27	73	182	256	721
Original analysis V4-V6 (5)^	55,163 (2,835 – 205,548)	28	60	82	191	581

*Due to technical issues when merging forward and reverse reads while using a pipeline that generates amplicon sequence variants (ASVs), only forward reads were used in updated bioinformatic processing.

^The original publication describes the sequencing read depth prior to bioinformatic processing. The number of classified reads are not listed, but were extracted from OTU tables provided from the PFDN for this updated analysis.

### Comparisons of Recovered Taxa in Original Versus Updated Analysis

**Figure 3:**
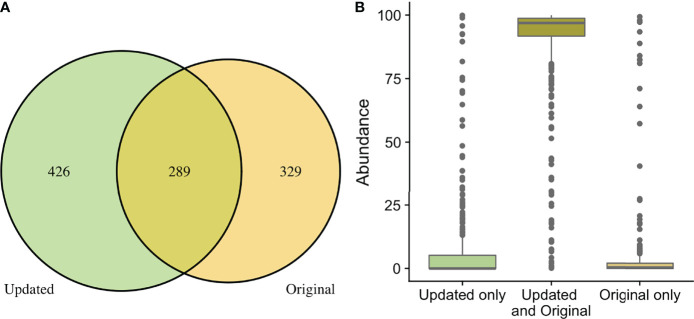
Comparison of taxa recovered through original and updated processing. OTU tables from the original study were obtained and compared to data generated from ASV tables after repeat processing. **(A)** depicts the number of individual genera that were identified after the original and updated processing pipelines. There were 329 and 432 unique genera identified with the original and updated pipelines, respectively. A total of 289 genera were identified with both processing pipelines. **(B)** shows the relative sequence abundances of those identified with only original or updated processing, as well as those identified with both pipelines. As depicted, the unique genera that are only identified in the original or updated pipelines tend to be low abundance sequences, while the large majority of the highly abundant sequences were identified through both processing pipelines.

When comparing the originally processed OTU table and updated ASV table from data generated by the V4 amplicon, 329 genera were only identified with original processing, 426 were only identified with updated processing, and 289 were overlapping and identified with both (**Figure 3A**). Though there were many non-overlapping genera, these were represented in the small proportion of the low abundance sequences from all samples (**Figure 3B**). The 329 genera that were uniquely identified with original processing are included in **Supplemental Table 2** and had counts ranging from 2-211. The 426 genera that were uniquely identified with updated processing are included in **Supplemental Table 3** and had counts ranging from 1-178. Of these, a total of 179 genera had a mean relative abundance <0.0005 and 66 genera had a mean relative abundance <0.0001, which were the filtering thresholds used in this analysis. Non-overlapping genera from original and updated processing with mean relative abundances >0.2 are displayed in **Table 2**.

**Table 2:** Unique taxa with highest mean abundances from original and updated processing.

	Genus	Count	Maximum Relative Abundance	Minimum Relative Abundance	Mean Relative Abundance
Original Processing	*Serratia*	200	95.8583359	0.00107388	2.67694661
	*Escherichia*	120	77.6694728	0.00072271	2.13110697
	*Clostridium*	210	82.1156486	0.00230984	0.6235074
	*Enterobacter*	108	43.4458227	0.00072271	0.23072134
Updated Processing	Escherichia/Shigella*	85	99.8775177	0.01175254	4.91545093
	*Cutibacterium*	178	58.8692498	0.01118443	3.94134097
	*Actinotignum*	53	64.7708383	0.00987882	0.62764323
	*Clostridium_sensu_stricto* ^^^	44	88.5600496	0.00498915	0.52225445
	*Proteus*	9	71.5686275	0.0122444	0.37338724
	*Ezakiella*	72	13.1103903	0.003139	0.22165684
	*Methylophilus*	67	4.84244259	0.00356837	0.21395235

Only taxa with mean relative abundances >0.2 listed here. For full lists of unique taxa from original and updated analyses, refer to **Supplemental Tables 2, 3**, respectively.

*The SILVA database classifies as Escherichia/Shigella while other databases (e.g., Greengenes in the original analysis) classify as Escherichia. Thus, this classification appears unique within original and updated datasets but could refer to similar genera.

^Classified as Clostridium_sensu_stricto in updated analysis using the SILVA database. It is unclear if this is a subset of Clostridium, or if this name in SILVA refers to the genus Clostridium from the Greengenes database.

### Comparisons of Recovered Taxa by Sequencing Amplicons

**Figure 4:**
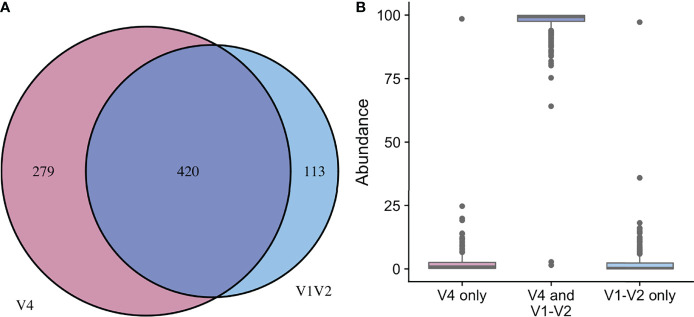
Comparison of taxa recovered from different sequencing amplicons. For samples with classified taxa from both V1-V2 and V4 regions (n=164), paired ASV tables were compared. **(A)** shows that 113 unique genera were identified with the V1-V2 region, 279 unique genera were identified with the V4 region, while 420 genera were shared and identified with both regions. **(B)** shows the relative sequence abundances of those identified with each region, as well as those that were identified with both regions. The unique genera that are only identified with one region are extremely low abundance sequences with an occasional outlier, while the large majority of the highly abundant sequences were identified in both regions (i.e., V1-V2 and V4 regions).

**Figure 5:**
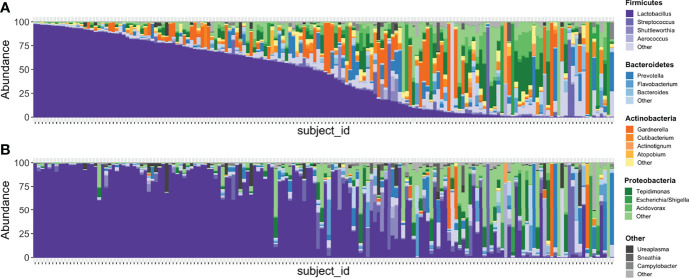
Stacked bar plots illustrating relative abundances of taxa in 167 samples with paired V1-V2 and V4 data. **(A)** depicts the taxa recovered with the V4 region while **(B)** depicts the taxa recovered with the V1-V2 region. Each vertical bar depicts an individual sample with plots aligned to compare recovery of data from the same sample in each amplicon. Phyla are assigned distinct colors (e.g., Firmicutes = purple, Bacteroidetes = blue, Actinobacteria = orange, Proteobacteria = green) with individual genera shaded differently. The most intense color shade within each phylum refers to the most abundant genus identified. Though many genera are recovered in similar abundances between both amplicons, *Gardnerella* is one that is noticeably different, with substantially more identified in sequencing data generated from the V4 region.

**Figure 6:**
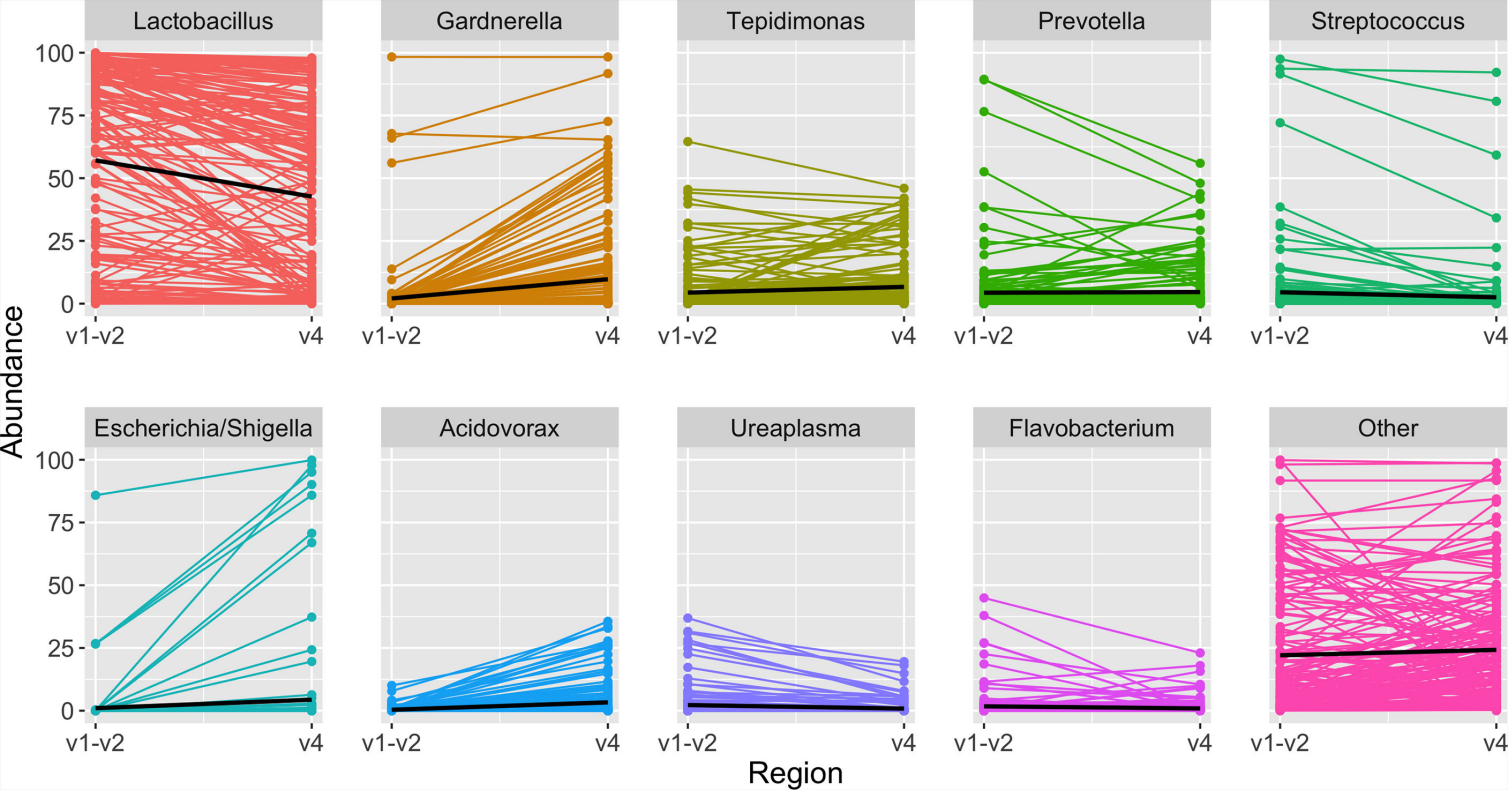
Highest abundance genera among paired samples. Each panel depicts the relative abundance of one genus. On the left is the relative abundance from the V1-V2 amplicon, connected by a line to the right, which shows the relative abundance in the same sample when identified from the V4 amplicon. In each panel the black line summarizes the median abundances across all paired samples. When comparing results from the same sample sequenced with two different amplicons, *Lactobacillus* was identified in slightly higher abundance with the V1-V2 amplicon, while other genera including *Gardnerella, Tepidomonas, Escherichia/Shigella*, and *Acidovorax* were identified in slightly higher abundance with the V4 amplicon.

In the updated analysis, a total of 164/207 (79%) of samples had paired classified taxa from V1-V2 and V4 regions (generated after sequencing V1-V3 and V4-V6 amplicons, respectively). Of these, 113 genera were only represented in the V1-V2 dataset, 279 were only represented in the V4 dataset, and 420 were overlapping and represented in both the V1-V2 and V4 datasets (**Figure 4A**). Like patterns detected when comparing OTU and ASV tables, the most abundant genera were overlapping and identified in both amplicons, while non-overlapping genera were identified in low abundance sequences (**Figure 4B**). Of the taxa that were represented in both V1-V2 and V4 regions, the median abundance was 99.3%. Of the taxa that were only represented in V1-V2, the median abundance was 0.35%; of the taxa that were only represented in V4, the median abundance was 0.88%. Taxa recovered per sample from V1-V2 and V4 regions are shown in **Figure 5**. Paired abundances from V1-V2 and V4 regions from the most highly abundant genera are summarized in **Figure 6**, with the remaining genera summarized in **Supplemental Figure 2**. A higher relative abundance of *Lactobacillus* was identified with the V1-V2 region, while slightly higher relative abundances of *Gardnerella, Tepidomonas, Escherichia/Shigella*, and *Acidovorax* were identified with the V4 region, with subtle differences in other genera. Without further testing and validation, it is unknown which of these two regions more accurately reflect true bacterial presence in the urinary bladder. However, multiple factors led us to infer that the V4 data might be more reliable in this dataset. First, in earlier stages of processing, it was noted that the V1-V2 region contained many sequences mapping to non-bacterial taxa (e.g., archaea, eukaryote, or not assigned) when compared to the SILVA reference database while the V4 region mapped mainly to bacterial taxa, as expected. Secondly, the V1-V2 region recovered *Gardnerella* in a sparser manner than the V4 region did. *Gardnerella* are biologically expected when reviewing prior urinary and vaginal microbiome data. Based on these considerations, we considered the V4 region data to be more reliable, and these data were selected for statistical analyses. This mirrors the original analysis, in which the authors elected to focus only on V4-V6 region sequencing results in their publication (Komesu et al., 2018).

### Testing for Associations Between Individual Taxa and Clinical Phenotypes

In BGCR analysis, we did not identify differences in microbial composition between MUI and control participants after adjusting for clinical covariates (PJAP = 0.337, indicating only a 33.7% probability of differences in the individual taxa).

### Clustering Into Bacterial Communities

**Figure 7:**
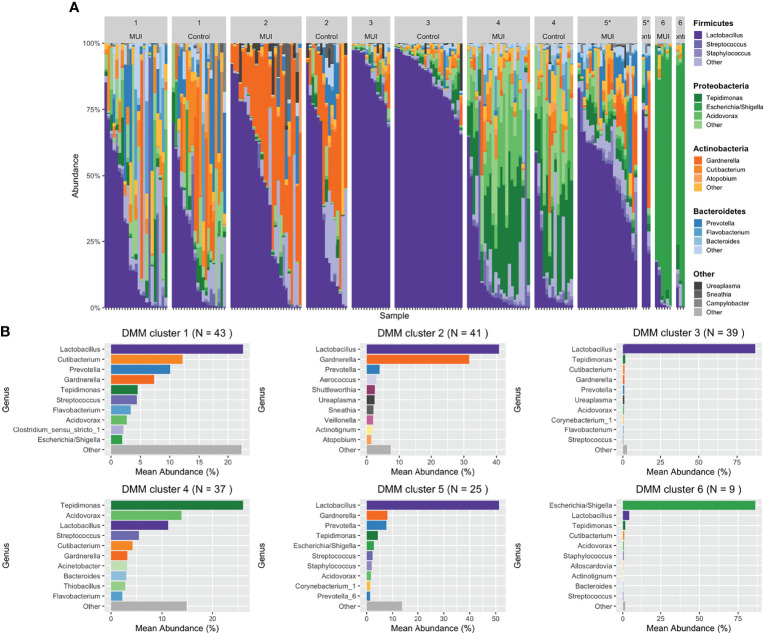
Stacked bar plots illustrating results from updated analysis when combining filtered ASVs from individual samples into bacterial communities using DMM clustering. For DMM clustering a total of 6 clusters were chosen *a priori*. Clusters are numbered with each cluster further organized by those samples originating from women with MUI versus control. **(A)** shows DMM clustering results with cluster membership that is significantly different between MUI and controls identified with an asterisk(*). **(B)** shows more detail about the relative abundances of various genera that contribute to each cluster.

As was done in the original analysis, we created a sub-structure within the microbial data by clustering samples into bacterial communities. We first repeated the original strategy using DMM modeling with the reprocessed data. In DMM modeling, the number of final clusters are pre-specified. Since the original analysis selected 6 clusters, we chose the same number for the updated analyses. **Figure 7** shows the 6 DMM clusters (i.e., bacterial communities) that we identified with filtered reprocessed data grouped by MUI and control phenotypes. We also clustered filtered reprocessed data using DTMM modeling where the number of clusters are mathematically chosen based on the data. Using the DTMM approach, there were only 3 clusters when filtering at 0.0005, though a 4^th^ cluster appeared when using a less stringent filtering threshold of 0.0001 (**Supplemental Figure 3**). This illustrates how multiple analysis steps, including the filtering strategy and clustering method could influence overall results, and thus should be carefully selected to best illustrate data without over-emphasizing “noise” within the dataset.

### Testing for Associations Between Bacterial Communities and MUI Versus Control Phenotype

Multivariable models were used to determine whether bacterial communities were associated with MUI versus control status, while controlling for other relevant covariates. We first created models that incorporated the clinical site. While some of the sites were significantly associated with outcomes, associations with clinical site were not stable among different models (see **Supplemental Information**). To avoid overfitting models, clinical site was removed in final models, which incorporated bacterial communities and the following covariates: age, smoking status, ethnicity, BMI, composite menopausal/hormonal status, vaginal pH, history of recurrent UTI, and number days from the most recent catheterization.

In our updated analysis using re-processed and filtered data as well as bacterial communities generated from DMM clustering, Cluster 5 was associated with MUI (p=0.03) with a trend towards cluster 3 being associated with controls (p=0.08). Cluster 5 refers to one with moderate *Lactobacilli* (mean relative abundance of ~50%), followed by almost equal *Gardernella* and *Prevotella* (mean relative abundances of 8.5-9%) with several other low abundance genera (see **Figure 7B**). Cluster 3 has much higher abundance of *Lactobacilli* (mean relative abundance almost 90%) with very small components of others (see **Figure 7B**). The covariates BMI and Latina ethnicity remained significantly associated with MUI, even when controlling for other variables, including bacterial community types.

The updated analyses that best approximates what was performed in the original analyses using unfiltered data is one where the least restrictive filtering threshold of 0.0001 is applied. Using this filtering threshold, DMM clustering was repeated giving 6 new bacterial communities, as depicted in **Supplemental Figure 4**. **Table 3** summarizes the results when the same model and clustering technique is used on data that are filtered differently. With a less restrictive threshold, Cluster 2 (p < 0.05) and Cluster 6 (p = 0.01), the latter with a composition similar to Cluster 5 above, were significantly associated with MUI while controlling for other covariates, including those that were also significantly associated with the MUI outcome, such as BMI (p < 0.01) and Latina ethnicity (p=0.02). In review of the actual taxa within clusters, it appears that the reference group in this model (Cluster 1) was characterized by very high abundances of *Lactobacilli* (see **Supplemental Figure 5**) and was the only group with a higher number of controls compared to MUI, despite the fact that control samples were under-represented in the overall dataset (~40% of overall samples).

**Table 3:** Updated analysis multivariable model testing for associations between MUI versus control.

Variable	Less restrictive filtering threshold (0.0001)p value	Conservative filtering threshold (0.0005)p value
Microbial community by DMM clustering		
Cluster 1	(reference)	
Cluster 2	0.045*	0.605
Cluster 3	0.674	0.079
Cluster 4	0.091	0.415
Cluster 5	0.400	0.030*
Cluster 6	0.010*	0.883
Age (years)	0.107	0.177
Latina Ethnicity	0.017*	0.023*
Body Mass Index (kg/m^2^)	0.002*	0.015*
Smoking Status	0.272	0.169
Vaginal pH	0.304	0.278
Menopause/Hormone Status^	0.403	0.626
Recurrent UTI	0.995	0.996
# days since prior catheterization	0.992	0.992

DMM (Dirichlet multinomial mixture); UTI (urinary tract infection)

*Significant association with MUI (mixed urinary incontinence)

^Composite variable of menopausal status and presence of hormone

A general inference from models incorporating bacterial communities is that communities with high proportions of *Lactobacilli* are associated with control status and communities with lower *Lactobacilli* and higher relative proportions of a combination of *Gardnerella and Prevotella* are associated with MUI status. Contrary to the original analysis, we did not perform subanalyses of participants age < 51 years and those 51 or older, but rather included the composite menopausal/hormonal status variable when modeling data. Our model also included history of recurrent UTIs, vaginal pH, and number of days since prior catheterization, since these are covariates that could contribute to further variability in urinary microbiome datasets.

We also performed multiple sensitivity analyses where we used the same modeling approach but with filtered data clustered with DTMM methods. DTMM generated fewer clusters (3 total) and we did not find signification associations between bacterial clusters generated with DTMM and the clinical phenotypes of MUI and control status. In these models, BMI and Latina ethnicity still remained significantly associated with MUI (p=0.007, p=0.004, respectively). Results are displayed in **Supplemental Figure 3**.

## Discussion

We re-analyzed previously generated sequencing data using updated bioinformatic techniques and refined the statistical analyses. This updated analysis offered several interesting nuances that enhance clinical inferences regarding the relationship between the urinary microbiome and MUI. In the original publication, researchers did not find differences in bacterial community types among women with MUI and controls, though a *post hoc* analysis found some associations between bacterial communities and MUI exclusively in women < 51 years of age. With our updated approach to the data, we first examined whether individual taxa might be drivers of differences between MUI and control phenotypes. Using BGCR analysis, we did not find this to be the case, as there was a low probability of differences in microbial composition between MUI and controls. However, similar to the original analysis, we assessed for how substructures within the microbial data (e.g., bacterial communities) might be associated with MUI versus control phenotypes. We were indeed able to confirm that associations between bacterial communities and MUI exist. However, after incorporating a variable that accounts for menopausal/hormone status in our model, we no longer found that associations differ by age. Even when sequencing data were filtered differently, associations between bacterial communities and MUI status remained robust with slightly different actual clusters (i.e., community members). This leads us to conclude that there is not likely to be one bacterial genus alone, but rather a difference in communities of bacteria, and perhaps how they interact, that is associated with mixed urinary incontinence phenotypes.

With this updated analysis we found that an updated bioinformatic processing pipeline recovers many different taxa compared to prior bioinformatic techniques. However, most of these differences exist in low abundance taxa that occupy a small proportion of the overall microbiome. We also confirmed that in urine, similar to other sample types, the region of the 16S rRNA gene that is chosen for sequencing can impact downstream results. For the most common (highest abundance) taxa, information will be recovered regardless of the bioinformatic strategy. However, less abundant taxa may have different biases based on the bioinformatics and sequencing amplicon chosen. For less abundant taxa, results may require additional validation and should be considered carefully when attempting to make inferences.

Strengths of our approach include the application of techniques that improve precision when analyzing low biomass samples. The bioinformatic processing pipeline applied in this study (i.e., DADA2) corrects for sequencing errors and chimeric sequences to improve accuracy. For updated processing we also used a different reference database (i.e., SILVA), since Greengenes, a database used in many prior urinary microbiome studies, has since been shown to have poor representation of bladder bacteria (Hoffman et al., 2021) and has not been updated since 2013. However, our bioinformatic approach is limited as specific expertise (e.g., knowledge of how to use R and other microbiome processing software like QIIME2^6^) may be required compared to prior “plug and play” approaches like the Illumina BaseSpace software. With enhancements in precision, we also encountered more data loss, as some samples did not have high enough quality sequencing information to provide taxonomic data. While we acknowledge that this may decrease the sample size, it may inherently be more scientifically rigorous to remove lower quality sequencing information. Despite technical differences in how sequencing data are handled, our updated processing identified a similar number of phyla and classes compared to the originally processed data (**Table 1**), with significantly more orders, families, and genera compared what was originally reported.

Another strength to our approach is that we tested multiple aspects of statistical analyses, including various filtering and clustering approaches, prior to arriving at our conclusions. Results from these sensitivity analyses offer insights to the urobiome community, as the filtering thresholds and clustering methodology chosen for a study may affect interpretation of overall results. Generally, researchers need to decide if they want to filter at a lower threshold, thereby keeping more sequencing data. With this approach, there is a risk of over-interpreting data in low biomass samples based on possible contaminants or low abundance sequence information. The other alternative is to filter at a higher threshold, which removes more data, but could result in missing an important association because clusters are less refined. This concept is illustrated in our study when evaluating multivariable models using DMM clustering to create microbial communities. In models with a less restrictive filtering threshold, there were associations that appeared statistically meaningful. When using the same clustering methodology with a more conservative filtering threshold, there are still statistically significant associations, but the clusters and downstream inferences are slightly different. Ultimately, it is only with repeated experiments and ongoing validation that we will expect to understand which approach best approximates the truth. However, it is important for investigators to understand how these choices that are made during statistical analyses may affect downstream results.

Existing groups are applying published techniques extrapolated from linear mathematical modeling to analyze microbial datasets. However, many of these techniques contain underlying assumptions of normally distributed data. High dimensional microbial datasets that are used in community-based analyses fail to meet these underlying assumptions, and thus additional techniques are being evaluated and developed. We had hypothesized that tree-based clustering approaches (e.g., DTMM) may be able to better resolve true signal from noise within a dataset. Compared to DMM clustering, DTMM puts more emphasis on lower abundance taxa when clustering. Incorporating a nonparametric mixture as in the available implementation of DTMM also avoids the often-difficult task of pre-specifying the number of clusters. While it was not the case that DTMM clustering was able to better resolve signal from noise in this analysis, it is still possible that other models more akin to machine learning may be useful in the future. For urinary microbiome data it is also not clear if the ratio of high to low abundance taxa (e.g., ratio of *Lactobacilli* compared to other Gram negative & anaerobic bacteria) is more biologically important or if individual low abundance taxa may be important. If the ratio of high abundance bacteria compared to all other bacteria is actually the most biologically important factor, then a clustering method such as DMM that emphasize the highest abundance taxa may actually be preferred.

Compared with the original analysis, we came to slightly different conclusions when evaluating results from our final multivariable models. While we agreed that there were associations between microbial communities and MUI, the context of these associations was different in our updated analysis. Specifically, in the original analysis, 17% of women reported their menopausal status as unknown prompting investigators to dichotomize age based on the approximate age of menopause (51 years) and analyze data in those less than 51 and those older than 51 years. With this approach there were different findings in the two sub-populations (Komesu et al., 2018), which is somewhat difficult to interpret. Furthermore, hormone status (e.g., whether oral or topical/vaginal hormones were used) was not incorporated into multivariable analyses despite differences noted in MUI and control populations. Multiple investigators have demonstrated that menopause and hormonal status affect microbial compositions in the vagina (Brotman et al., 2014; Gliniewicz et al., 2019), and we are now learning that these variables are associated with differences in microbial compositions of the bladder as well (Thomas-White et al., 2020). As such, the original clinical data were reviewed to assess how these data were obtained. In this process, we discovered that menopausal information was obtained twice, with one group of questions having more reliable response options. Furthermore, two clinicians (NYS and LB) reviewed all age, menopause, and hormone usage information. Using a combination of these responses, we were able to reliably create a composite variable that incorporated menopausal & hormonal information in an accurate manner. In addition to this composite variable, additional variables that could also confound microbial information such as vaginal pH, history of recurrent UTI, and number of days from prior catheterization were also incorporated into multivariable models, while they were not previously. With this modeling strategy, we no longer see age as a separate independent factor affecting microbial community types. Regardless of the modeling strategy used, multiple covariates remained associated with the bladder outcome of MUI, highlighting the importance of incorporating covariates into analyses of microbial data.

A limitation in our updated analysis is that we had to rely on previously generated sequencing information and were not able to influence laboratory aspects of the study. For example, the choice of using the V4-V6 amplicon with very short overlapping sequences resulted in the inability to merge forward and reverse sequencing reads during bioinformatic processing to create final reads with longer length. While the V1-V3 region had more overlapping sequence between forward and reverse reads, there were still similar issues in attempting to merge reads that would have resulted in substantial data loss. It is not clear how these issues were managed in the original analysis when using the Illumina BaseSpace Metagenomics App, which is a “black box” bioinformatics approach. Ultimately, for the updated analysis we chose to use forward reads only. When comparing the taxa recovered, both based on numbers of taxa classified (**Table 1**), as well as comparisons of the classifications between original and updated analyses (**Figure 3**), we have inferred that our analysis of forward read only data very closely approximates the information provided in the original analysis, which was stated to use merged reads.

Another limitation is that any sequencing method that uses shorter lengths of DNA (e.g., what occurs with one or two variable regions of the 16S rRNA gene) could result in some difficulty classifying sequences at higher resolutions such as genus or species. Thus, newer techniques that incorporate highly accurate long-read sequencing methods (Callahan et al., 2019; Callahan et al., 2021) may be helpful to characterize the microbiome in a new niche. We were limited to using previously generated sequencing data and thus could not take such steps to enhance accuracy and resolution of the dataset, which might prove to be useful. Also related to the goal of enhancing accuracy, many studies will now incorporate a mock microbial community with serial dilutions to allow for the application of additional methods of removing contaminant ASVs during bioinformatic processing (Davis et al., 2018; Karstens et al., 2019). Since the sequencing data used in this study were developed prior to large scale incorporation of this approach, a mock microbial community was not used. Given that urine is a low biomass sample type that may be influenced by contaminants, future studies would likely benefit from the incorporation of current methods to remove contaminant ASVs during bioinformatic processing. This strategy may also facilitate the ability to use less stringent filtering thresholds since many contaminants will have already been removed. However, Cao *et al.* recently provided data that bioinformatic contaminant removal and filtering are complementary methods and should be employed together in highly rigorous studies (Cao et al., 2020).

With the continued evolution of computational techniques, we expect further improvements and guidelines for analyzing microbial datasets. With this updated analysis, we offer additional insights for investigators embarking on urinary microbiome analyses, and also enhanced clinical inferences regarding the relationship between the urinary microbiome and MUI. Specific considerations should be given to the amplicon (i.e., region of 16S rRNA gene) chosen, the bioinformatic processing pipeline, and the reference database that is used to ensure that updated resources containing adequate representation of urinary microbiota are used. Though default filtering thresholds and clustering methodologies exist, these parameters may need to be optimized based on the questions that are being posed in a microbial dataset. Finally, regardless of how analyses are conducted, multivariable analyses that incorporate potentially confounding clinical variables remain extremely important in analyses of microbial datasets.

## Data Availability Statement

Unprocessed sequencing files are publicly shared on the Sequence Read Archive (SRA), Bioproject ID 703967, Accession #: PRJNA703967. Further inquiries can be directed to the corresponding author.

## Ethics Statement

This re-analysis of sequencing data from human participants was reviewed and approved by the Duke University Institutional Review Board (Pro #00102155). The patients/participants provided their written informed consent at the time of inclusion in the original HMS-ESTEEM study.

## Author Contributions

All authors have made substantial contributions as follows: conception or design of the work; or the acquisition, analysis or interpretation of data for the work (NS, LM, LB, JM, CH, ED, ZW, LK) drafting the work or revising it critically for important intellectual content (NS, LM, LB, LK) provide approval for publication of the content (NS, LM, LB, JM, CH, ED, ZW, LK) agree to be accountable for all aspects of the work in ensuring that questions related to the accuracy or integrity of any part of the work are appropriately investigated and resolved (NS).

## Funding

K01 DK116706 (LK, Career Development Award) R01 GM135440 (LM & NS, method development for microbiome data). American Association of Obstetricians and Gynecologists Foundation (AAOGF) Bridge Funding Award (NS).

## Conflict of Interest

The authors declare that the research was conducted in the absence of any commercial or financial relationships that could be construed as a potential conflict of interest.

## Publisher’s Note

All claims expressed in this article are solely those of the authors and do not necessarily represent those of their affiliated organizations, or those of the publisher, the editors and the reviewers. Any product that may be evaluated in this article, or claim that may be made by its manufacturer, is not guaranteed or endorsed by the publisher.

## Footnotes

1
https://greengenes.secondgenome.com/

2
https://twbattaglia.gitbooks.io/introduction-to-qiime/content/lefse.html

3
https://pfdnetwork.azurewebsites.net/

4
https://www.ncbi.nlm.nih.gov/sra

5
https://github.com/MaStatLab/DTMM

6
https://qiime2.org/
