# Supplementary material for: Updating Urinary Microbiome Analyses to Enhance Biologic Interpretation
Source: Front Cell Infect Microbiol. 2022 Jul 8;12:789439. doi: 10.3389/fcimb.2022.789439 (PMC9309214; doi:10.3389/fcimb.2022.789439)
Supplement: Supplementary file 1 [file DataSheet_1.pdf]

**Supplemental Information**

Supplemental Table 1: Methods and variables for multivariable modeling

| | **Original Analysis** | **Updated analysis** |
|---|---|---|
| Variable Selection Method | …"demographic and medical history variables significantly associated with DMM community type in bivariate analysis ($p<0.05$) and variables associated with MUI versus control and other variables of clinical significance" were selected as covariates.(1) | All clinical variables collected in the HMS-ESTEEM study(2, 3) were ranked^ based on *a priori* knowledge of how each might be associated with microbial community type or MUI. Highest tier of ranked clinical variables were incorporated into multivariable models; certain variables without associations were removed to avoid overfitting models (i.e., avoid exceeding 15 degrees of freedom). |
| Clinical Variables | | |
| Age | Included; due to associations between age and DMM communities, reduced models in age <51 and age >51 created | Included; reduced models not necessary as associations no longer remain when incorporating other covariates |
| Race* | Not included | Tier 1 variable evaluated in preliminary models; not included in final model (lack of association) |
| Ethnicity (Latina vs not Latina)* | Included | Included |
| BMI | Included | Included |
| Recurrent UTI (3 or more in prior year)* | Not included despite associations with MUI status in bivariate analysis | Included |
| Smoking Status (active vs not active) | Included | Included |
| Menopausal Status | Not included | Included as composite variable[&] |
| Hormone Status | Not included despite associations with MUI status in bivariate analysis | Included as composite variable[&] |
| Vaginal pH | Not included | Included |
| Number days since prior catheterization | Not included | Included[$] |

| | | |
|---|---|---|
| Technical Variables | | |
| Clinical Site | Model "included a random effect for clinical site."(1) | Tier 2 variable included in preliminary models; removed from final model due to inconsistent associations and to avoid overfitting models. |
| Sample processing time | Not included | Not included[#] |

DMM = Dirichlet multinomial mixture, HMS-ESTEEM = Human Microbiome Study in the Effects of Surgical Treatment Enhanced with Exercise for Mixed Urinary Incontinence randomized trial, MUI = mixed urinary incontinence, BMI = body mass index, UTI = urinary tract infection
^Variables ranked into three tiers with Tier 1 comprising 10 variables listed above with highest likelihood for having associations with urinary microbes and/or clinical condition. Tier 2 variables included parity, responses on validated questionnaires, clinical site, and presence of detrusor overactivity on urodynamic testing; Tier 3 variables were all others.
*Based on patient self-report
[&]Menopausal status (e.g., premenopausal, postmenopausal, unknown) was collected but 17% responded as "unknown". Thus, this variable was not included in initial HMS-ESTEEM analysis. Upon detailed review, clinicians were able to resolve the "unknown" responses by considering 3 variables in context with each other: age, menopausal status, and use of estrogen hormone. Thus, a composite variable was derived that incorporates both menopausal & hormonal status with 3 potential responses: 1) postmenopausal, no hormones; 2) post-menopausal any hormones (oral, transdermal, vaginal); 3) premenopausal.
[$]Number of days since prior catheterization was assessed in preliminary models as both a continuous variable and as a categorical variable based on clinical assessment of the level of risk of modifying urinary microbial communities (<30 days; 30-90 days; >90 days).
[#]8 samples with prolonged processing time qualitatively explored. Samples were processed 4-6 days after collection due to weekend shipping (compared to within 2 days per study protocol). All were maintained and shipped with DNA protectant (Assay Assure™) and no differences were noted in data quality.

Supplemental Table 2: Taxa identified in original analysis but not with updated analysis

| Genus | Count | Maximum Relative Abundance | Minimum Relative Abundance | Mean Relative Abundance |
|---|---|---|---|---|
| Pediococcus | 211 | 2.22222222 | 0.00182802 | 0.16989838 |
| Clostridium | 210 | 82.1156486 | 0.00230984 | 0.6235074 |
| Alkalibacillus | 207 | 0.16841132 | 0.00172746 | 0.03570585 |
| Planifilum | 205 | 0.15757535 | 0.00141247 | 0.02677427 |
| Cohnella | 204 | 0.2265778 | 0.00086373 | 0.03417892 |
| Pseudoalteromonas | 201 | 0.28975009 | 0.0009171 | 0.02126797 |
| Serratia | 200 | 95.8583359 | 0.00107388 | 2.67694661 |
| Thiomonas | 195 | 0.9578975 | 0.00060928 | 0.11132352 |
| Nocardia | 190 | 0.3742925 | 0.00098385 | 0.02157453 |
| Desulfosarcina | 186 | 0.32575997 | 0.00080278 | 0.01013734 |
| Lentibacillus | 183 | 0.04730476 | 0.00086373 | 0.01084026 |
| Fructobacillus | 180 | 0.07303268 | 0.00077267 | 0.01101296 |
| Pseudidiomarina | 180 | 0.41651576 | 0.00053694 | 0.0138923 |
| Tetragenococcus | 180 | 0.0516723 | 0.00074138 | 0.00870449 |
| Actinocatenispora | 177 | 1.82017671 | 0.00091291 | 0.0196844 |

| | | | | |
|---|---|---|---|---|
| Candidatus Blochmannia | 176 | 0.49382716 | 0.0004865 | 0.02143128 |
| Serinicoccus | 174 | 0.5643739 | 0.00080278 | 0.03461431 |
| Virgibacillus | 173 | 0.03788662 | 0.00126488 | 0.00628113 |
| Gluconobacter | 162 | 0.13647845 | 0.00050648 | 0.01541737 |
| Desulfuromusa | 153 | 0.05056167 | 0.0004865 | 0.00521716 |
| Candidatus Phytoplasma | 148 | 1.41285097 | 0.0004865 | 0.03381678 |
| Glycomyces | 148 | 0.02653341 | 0.00091291 | 0.00375915 |
| Marinococcus | 143 | 0.04255036 | 0.00050648 | 0.00299181 |
| Rhodocyclus | 143 | 0.13669351 | 0.00080278 | 0.01151565 |
| Oxalobacter | 142 | 2.46337105 | 0.00077267 | 0.12387415 |
| Sebaldella | 141 | 0.46365185 | 0.00060928 | 0.01326546 |
| Halanaerobium | 137 | 1.03583538 | 0.00050648 | 0.01947472 |
| Chlorobaculum | 136 | 0.14337871 | 0.00053694 | 0.00607068 |
| Candidatus Amoebophilus | 135 | 0.45772993 | 0.00053694 | 0.0135186 |
| Oenococcus | 135 | 0.56799512 | 0.00091291 | 0.0083796 |
| Alkaliphilus | 134 | 1.07339667 | 0.00050648 | 0.01959516 |
| Rickettsia | 134 | 1.07035276 | 0.0004865 | 0.01442643 |
| Vagococcus | 133 | 0.59760956 | 0.00101895 | 0.00952868 |
| Giesbergeria | 132 | 0.8904941 | 0.000574 | 0.01982739 |
| Helicobacter | 131 | 0.03698635 | 0.0004865 | 0.00324713 |
| Borrelia | 128 | 0.83492127 | 0.00050648 | 0.02221006 |
| Actinopolymorpha | 127 | 0.19138125 | 0.00077267 | 0.00609915 |
| Desulfosporosinus | 127 | 0.02786615 | 0.00050648 | 0.00297469 |
| Listeria | 126 | 0.20545315 | 0.00050648 | 0.00744422 |
| Amycolatopsis | 124 | 0.10962102 | 0.0004865 | 0.00448506 |
| Caldicellulosiruptor | 124 | 0.33628609 | 0.0004865 | 0.00673449 |
| Syntrophomonas | 124 | 0.02293613 | 0.00050648 | 0.00271 |
| Burkholderia | 123 | 1.41033029 | 0.0004865 | 0.02749692 |
| Chromatium | 123 | 0.03527337 | 0.0004865 | 0.00288414 |
| Escherichia | 120 | 77.6694728 | 0.00072271 | 2.13110697 |
| Sinorhizobium | 120 | 0.15371349 | 0.0004865 | 0.01257272 |
| Lewinella | 119 | 1.65598862 | 0.00050648 | 0.04756113 |
| Microvirus | 118 | 0.07054674 | 0.0004865 | 0.00375738 |
| Planococcus | 118 | 0.18820348 | 0.00076326 | 0.00421161 |
| Thiocapsa | 118 | 0.01810938 | 0.00053694 | 0.00206633 |
| Coraliomargarita | 117 | 1.59484371 | 0.0004865 | 0.05908499 |
| Sporosarcina | 116 | 3.01253837 | 0.00053694 | 0.02025187 |
| Luteococcus | 115 | 2.3319895 | 0.0004865 | 0.03734356 |
| Acetobacterium | 114 | 0.10566949 | 0.0004865 | 0.00623854 |
| Actinopolyspora | 113 | 0.02851169 | 0.0004865 | 0.00225367 |

| | | | | |
|---|---|---|---|---|
| Runella | 113 | 2.30036018 | 0.00050648 | 0.04467686 |
| Tsukamurella | 112 | 0.13138078 | 0.00053694 | 0.00354181 |
| Erysipelothrix | 111 | 1.57061463 | 0.00050648 | 0.01523398 |
| Caloramator | 109 | 0.68055639 | 0.00050648 | 0.0141222 |
| Saccharomonospora | 109 | 0.08451942 | 0.00074138 | 0.00517871 |
| Agromyces | 108 | 1.31395754 | 0.00053694 | 0.013373 |
| Calothrix | 108 | 10.7953462 | 0.00053694 | 0.15104215 |
| Enterobacter | 108 | 43.4458227 | 0.00072271 | 0.23072134 |
| Nitrosococcus | 108 | 0.40738362 | 0.00074138 | 0.00504142 |
| Olivibacter | 108 | 0.85715726 | 0.00050648 | 0.01897427 |
| Pilimelia | 108 | 0.0803976 | 0.0004865 | 0.00223557 |
| Caldilinea | 107 | 0.31080748 | 0.0004865 | 0.00801449 |
| Actinomycetospora | 106 | 0.03885398 | 0.00072271 | 0.00207082 |
| Desulfonatronum | 104 | 0.21142211 | 0.0004865 | 0.00496187 |
| Pelagicoccus | 104 | 0.37280048 | 0.000574 | 0.00806247 |
| Jiangella | 103 | 0.20908573 | 0.00086373 | 0.00587544 |
| Rhodothermus | 102 | 0.10856435 | 0.00076326 | 0.00517386 |
| Tenacibaculum | 102 | 1.85106431 | 0.00111956 | 0.0463644 |
| Roseivivax | 101 | 0.01242668 | 0.00053694 | 0.00178773 |
| Geobacter | 100 | 1.95290642 | 0.00074138 | 0.02606747 |
| Providencia | 94 | 2.40854763 | 0.0004865 | 0.01491454 |
| Dolichospermum | 93 | 0.12157948 | 0.000574 | 0.00219961 |
| Tetrasphaera | 91 | 0.03957001 | 0.00086373 | 0.0031905 |
| Rathayibacter | 88 | 0.32556809 | 0.00077267 | 0.01057361 |
| Halanaerobacter | 87 | 0.01540055 | 0.00077267 | 0.00162983 |
| Propionispora | 87 | 0.62544373 | 0.0004865 | 0.0116889 |
| Kushneria | 86 | 0.20353284 | 0.00074138 | 0.00367123 |
| Gallionella | 85 | 0.07981908 | 0.00050648 | 0.00281381 |
| Rhodospirillum | 84 | 0.4815207 | 0.0004865 | 0.00717818 |
| Sulfobacillus | 84 | 3.21194351 | 0.00053694 | 0.05992868 |
| Heliorestis | 82 | 0.05314786 | 0.0004865 | 0.00263832 |
| Marinomonas | 82 | 0.0352959 | 0.00072271 | 0.00204954 |
| Pectinatus | 82 | 0.82880624 | 0.0004865 | 0.00500994 |
| Streptosporangium | 81 | 0.17636684 | 0.00091291 | 0.00268521 |
| Desulfobacter | 80 | 0.02956262 | 0.0004865 | 0.00161285 |
| Candidatus Liberibacter | 79 | 0.86509837 | 0.00074138 | 0.01141744 |
| Acholeplasma | 78 | 0.35032319 | 0.00076326 | 0.00607285 |
| Chromobacterium | 78 | 0.12713588 | 0.00050648 | 0.00333997 |
| Cystobacter | 78 | 0.25862943 | 0.00050648 | 0.01062787 |
| Carboxydocella | 76 | 0.02771212 | 0.0004865 | 0.00101757 |

| | | | | |
|---|---|---|---|---|
| Glaciecola | 76 | 0.19685039 | 0.000574 | 0.00209491 |
| Desulfurispirillum | 74 | 0.01450356 | 0.0004865 | 0.00100745 |
| Euzebya | 74 | 0.01994449 | 0.00060928 | 0.00149015 |
| Moorella | 73 | 0.39414234 | 0.00076326 | 0.00314505 |
| Yaniella | 73 | 0.03865741 | 0.00053694 | 0.00120071 |
| Caldithrix | 72 | 0.12534414 | 0.00050648 | 0.00242648 |
| Rubritalea | 71 | 0.13861474 | 0.00077267 | 0.00370059 |
| Viridibacillus | 71 | 0.01489913 | 0.0004865 | 0.00098694 |
| Actinomadura | 70 | 0.01196729 | 0.00060928 | 0.00105139 |
| Trichodesmium | 70 | 0.31498953 | 0.00091361 | 0.00282673 |
| Acidimicrobium | 69 | 0.20255216 | 0.00050648 | 0.00316594 |
| Gillisia | 69 | 0.12615975 | 0.00050648 | 0.0025137 |
| Candidatus Scalindua | 68 | 0.89845382 | 0.00060928 | 0.0116694 |
| Desulfotomaculum | 68 | 0.29190399 | 0.00060928 | 0.00270288 |
| Anaerobranca | 67 | 0.07547821 | 0.00077267 | 0.00253532 |
| Demequina | 67 | 0.34297644 | 0.00050648 | 0.0062782 |
| Pontibacillus | 67 | 0.03089076 | 0.00050648 | 0.0009824 |
| Thermoanaerobacterium | 67 | 0.0199409 | 0.0004865 | 0.00114046 |
| Tolumonas | 67 | 0.28975009 | 0.0004865 | 0.00537887 |
| Candidatus Rhabdochlamydia | 66 | 0.43175336 | 0.00053694 | 0.0141035 |
| Thiohalorhabdus | 65 | 0.01810938 | 0.00076326 | 0.00118874 |
| Melissococcus | 63 | 0.07237826 | 0.0004865 | 0.00132001 |
| Marinitoga | 62 | 0.11381609 | 0.000574 | 0.00268015 |
| Thermogemmatispora | 61 | 0.22101361 | 0.00086373 | 0.00253155 |
| Anaerobacillus | 60 | 0.0100842 | 0.0004865 | 0.00067889 |
| Mesoplasma | 60 | 0.4577532 | 0.00093628 | 0.00359135 |
| Sporolactobacillus | 60 | 0.2756129 | 0.0004865 | 0.00492195 |
| Thermoanaerobacter | 60 | 0.0665155 | 0.0004865 | 0.00138971 |
| Candidatus Rhodoluna | 59 | 2.12413546 | 0.00121972 | 0.07719623 |
| Thermobaculum | 59 | 0.24172555 | 0.00050648 | 0.00569034 |
| Thioalkalimicrobium | 59 | 0.01499644 | 0.00053694 | 0.00098397 |
| Cyanobacterium | 58 | 0.02126302 | 0.00076326 | 0.0010967 |
| Ectothiorhodospira | 57 | 0.02771212 | 0.00050648 | 0.00131042 |
| Leucobacter | 57 | 0.07037114 | 0.00072271 | 0.00229653 |
| Ramlibacter | 57 | 1.26363613 | 0.00053694 | 0.03477767 |
| Desulfonatronovibrio | 56 | 0.03527337 | 0.000574 | 0.00072201 |
| Marichromatium | 56 | 0.03527337 | 0.00077267 | 0.00118351 |
| Petrotoga | 55 | 0.00711961 | 0.00077267 | 0.00069954 |
| Segetibacter | 55 | 0.28587099 | 0.00076326 | 0.00736039 |

| | | | | |
|---|---|---|---|---|
| Natronincola | 54 | 0.76547742 | 0.00053694 | 0.0119956 |
| Aquitalea | 53 | 0.0158831 | 0.0004865 | 0.00061441 |
| Dokdonella | 52 | 0.13790726 | 0.00053694 | 0.00259077 |
| Polaribacter | 51 | 0.02752336 | 0.000574 | 0.0013033 |
| Flammeovirga | 50 | 0.05772122 | 0.00098385 | 0.00145223 |
| Francisella | 50 | 0.07054674 | 0.00060928 | 0.0012432 |
| Uliginosibacterium | 50 | 0.06277149 | 0.00074138 | 0.00164561 |
| Waddlia | 50 | 0.45566446 | 0.00100781 | 0.0076698 |
| Edaphobacter | 49 | 0.09000424 | 0.00072271 | 0.00235264 |
| Gramella | 49 | 0.02535583 | 0.00104386 | 0.00091341 |
| Methylonatrum | 49 | 0.0317662 | 0.00074138 | 0.00075013 |
| Ruegeria | 49 | 0.10206449 | 0.00093628 | 0.00222437 |
| Symbiobacterium | 49 | 0.01443894 | 0.00050648 | 0.00060738 |
| Stenoxybacter | 48 | 0.72520536 | 0.0004865 | 0.00474361 |
| Candidatus Methylacidiphilum | 47 | 0.07207476 | 0.00076326 | 0.00099077 |
| Thalassospira | 47 | 0.46330984 | 0.000574 | 0.0041046 |
| Dethiosulfovibrio | 46 | 4.78613622 | 0.0004865 | 0.02552911 |
| Actinokineospora | 44 | 0.00566134 | 0.0004865 | 0.00045573 |
| Litoricola | 44 | 0.06157119 | 0.00099497 | 0.00140654 |
| Curtobacterium | 43 | 0.07231321 | 0.00101709 | 0.00166664 |
| Salinibacterium | 43 | 0.25035598 | 0.00053694 | 0.0039407 |
| Ehrlichia | 42 | 1.57494763 | 0.00093628 | 0.0120104 |
| Kitasatospora | 42 | 0.02117747 | 0.00053694 | 0.00075425 |
| Dactylosporangium | 41 | 1.14771263 | 0.00110892 | 0.00929431 |
| Prauserella | 41 | 0.00739727 | 0.0004865 | 0.00042733 |
| Propionigenium | 41 | 0.01429422 | 0.00076326 | 0.00063513 |
| Kutzneria | 40 | 0.00401792 | 0.00053694 | 0.00037502 |
| Nonomuraea | 40 | 0.00994135 | 0.0004865 | 0.00044929 |
| Desulfomonile | 39 | 0.19970741 | 0.00072271 | 0.00268216 |
| Phenylobacterium | 39 | 0.18208108 | 0.00053694 | 0.00471549 |
| Rubrivivax | 39 | 0.54769512 | 0.0004865 | 0.01078394 |
| Sejongia | 39 | 0.86885966 | 0.00109867 | 0.01888471 |
| Arthrospira | 38 | 4.14984922 | 0.00091291 | 0.0242256 |
| Kaistobacter | 38 | 1.31708153 | 0.00053694 | 0.01674008 |
| Arthronema | 37 | 0.00626318 | 0.000574 | 0.0004266 |
| Bartonella | 37 | 0.04102996 | 0.00060928 | 0.00066936 |
| Tepidanaerobacter | 37 | 0.01966117 | 0.00093628 | 0.00081264 |
| Trabulsiella | 37 | 0.42963066 | 0.00097301 | 0.00773755 |
| Anaerolinea | 36 | 0.14298599 | 0.00050648 | 0.00304028 |

| | | | | |
|---|---|---|---|---|
| Acidithiobacillus | 35 | 0.0074028 | 0.0004865 | 0.00044267 |
| Arsenophonus | 35 | 0.07243752 | 0.00076326 | 0.00085991 |
| Haloanella | 35 | 0.05240204 | 0.00076326 | 0.0017964 |
| Oscillatoria | 34 | 0.01512681 | 0.00072271 | 0.00050506 |
| Salinivibrio | 34 | 0.03027012 | 0.0004865 | 0.00099961 |
| Azoarcus | 33 | 0.01656212 | 0.00077267 | 0.00054226 |
| Desulfacinum | 33 | 0.06710409 | 0.00098118 | 0.00090719 |
| Hyphomonas | 33 | 0.14718638 | 0.00086373 | 0.00105595 |
| Oleomonas | 33 | 1.10065515 | 0.00053694 | 0.03621413 |
| Erwinia | 32 | 0.79689557 | 0.00060928 | 0.0154089 |
| Nostoc | 32 | 0.01426086 | 0.0004865 | 0.00034046 |
| Pasteurella | 32 | 1.09372293 | 0.00076326 | 0.01043545 |
| Thermodesulfovibrio | 32 | 0.03279441 | 0.00099497 | 0.00070017 |
| Kineosporia | 31 | 0.0768935 | 0.00107388 | 0.00178475 |
| Microbulbifer | 31 | 0.00714711 | 0.00050648 | 0.00035287 |
| Thioalkalivibrio | 31 | 0.0082021 | 0.00076326 | 0.00040676 |
| Candidatus Glomeribacter | 30 | 0.00869036 | 0.00091291 | 0.00032628 |
| Candidatus Regiella | 30 | 0.03527337 | 0.00050648 | 0.00044834 |
| Methylibium | 30 | 0.40640849 | 0.00074138 | 0.00313861 |
| Methylosinus | 30 | 0.31952866 | 0.00091291 | 0.00327984 |
| Telmatospirillum | 30 | 1.46638328 | 0.00077267 | 0.00810093 |
| Leptospira | 29 | 0.11493084 | 0.000574 | 0.00099712 |
| Maricaulis | 29 | 0.00655888 | 0.00060928 | 0.0002473 |
| Psychroflexus | 29 | 0.06575024 | 0.00100781 | 0.00143932 |
| Rikenella | 29 | 0.41825051 | 0.00074138 | 0.00881474 |
| Sporotomaculum | 29 | 0.07044588 | 0.00117202 | 0.00082509 |
| Ammonifex | 28 | 0.0093129 | 0.00050648 | 0.00034391 |
| Amphritea | 28 | 0.0164042 | 0.00077267 | 0.00041436 |
| Desulfonauticus | 28 | 0.02945429 | 0.00091291 | 0.00044386 |
| Roseospira | 27 | 0.59299942 | 0.00097301 | 0.0080788 |
| Streptacidiphilus | 27 | 0.0053188 | 0.00098775 | 0.00027831 |
| Agrobacterium | 26 | 0.63492064 | 0.00098775 | 0.00608862 |
| Candidatus Endobugula | 26 | 0.01813927 | 0.00076326 | 0.00046236 |
| Geotoga | 26 | 0.00559779 | 0.00072271 | 0.00028801 |
| Klebsiella | 26 | 1.83194573 | 0.00060928 | 0.01171006 |
| Parascardovia | 26 | 1.92112931 | 0.00115493 | 0.0204805 |
| Sediminibacillus | 26 | 0.00472032 | 0.00086373 | 0.00024305 |
| Syntrophobacter | 26 | 0.13444146 | 0.000574 | 0.00149785 |
| Alcanivorax | 25 | 0.03976538 | 0.000574 | 0.00054557 |
| Candidatus Tammella | 25 | 0.0106754 | 0.0004865 | 0.00033561 |

| | | | | |
|---|---|---|---|---|
| Chondromyces | 25 | 0.77982844 | 0.00086373 | 0.00732268 |
| Chroococcus | 25 | 0.39386051 | 0.0004865 | 0.00363608 |
| Kosmotoga | 25 | 0.0082021 | 0.00098118 | 0.00031792 |
| Niabella | 25 | 0.49369577 | 0.00101709 | 0.00403888 |
| Psychromonas | 25 | 0.00937277 | 0.00060928 | 0.00032545 |
| Zhihengliuella | 25 | 0.14892627 | 0.00091361 | 0.00125719 |
| Brevibacillus | 24 | 0.01012453 | 0.00101493 | 0.00034989 |
| Cryocola | 24 | 0.45832315 | 0.0015649 | 0.00867527 |
| Avibacterium | 23 | 0.03479471 | 0.00112477 | 0.00067309 |
| Brenneria | 23 | 0.03538897 | 0.00098385 | 0.00098922 |
| Coprobacillus | 23 | 0.38319972 | 0.0017806 | 0.00510184 |
| Desulfuromonas | 23 | 0.04369968 | 0.00098775 | 0.00050651 |
| Fervidobacterium | 23 | 0.00730327 | 0.00080278 | 0.00024406 |
| Frankia | 23 | 0.02884557 | 0.00091361 | 0.00044791 |
| Pelotomaculum | 23 | 0.18174635 | 0.00074138 | 0.00127688 |
| Fibrobacter | 22 | 0.00475195 | 0.00050648 | 0.00024233 |
| Leptolyngbya | 22 | 0.07259289 | 0.0004865 | 0.00100115 |
| Mycoplana | 22 | 0.27847695 | 0.00147813 | 0.0051213 |
| Cryobacterium | 21 | 0.05070565 | 0.00093628 | 0.00063188 |
| Dickeya | 21 | 0.02771811 | 0.00121856 | 0.00054551 |
| Phaeobacter | 21 | 0.03515203 | 0.00072271 | 0.00052798 |
| Thermodesulfatator | 21 | 0.00296648 | 0.00074138 | 0.00016347 |
| Halorhodospira | 20 | 0.00714711 | 0.00097301 | 0.00025787 |
| Marinospirillum | 20 | 0.01905896 | 0.00099497 | 0.0002617 |
| Muricauda | 20 | 0.08568521 | 0.00112462 | 0.00086814 |
| Rhodovulum | 20 | 0.03097082 | 0.00074138 | 0.00073233 |
| Allochromatium | 19 | 0.1170135 | 0.0004865 | 0.00086892 |
| Desulfofrigus | 19 | 0.09538453 | 0.00111914 | 0.00086644 |
| Ferrimicrobium | 19 | 0.49589929 | 0.00101895 | 0.00298466 |
| Ancylobacter | 18 | 0.06502102 | 0.00077267 | 0.00088435 |
| Azorhizobium | 18 | 0.07227255 | 0.00098775 | 0.00154388 |
| Coriobacterium | 18 | 0.25412961 | 0.00076326 | 0.00146432 |
| Mechercharimyces | 18 | 0.10089596 | 0.00053694 | 0.00076139 |
| Nitrosovibrio | 18 | 0.12034901 | 0.00103335 | 0.0027115 |
| Pigmentiphaga | 18 | 0.00842389 | 0.00076326 | 0.00022347 |
| Rhizobium | 18 | 0.0772512 | 0.00098118 | 0.00107509 |
| Actinoallomurus | 17 | 0.00348068 | 0.000574 | 0.00016207 |
| Bellilinea | 17 | 0.01230553 | 0.00053694 | 0.00028594 |
| Hydrocoleum | 17 | 0.02858845 | 0.0004865 | 0.00044112 |
| Microcoleus | 17 | 0.05241215 | 0.000574 | 0.00049172 |

| | | | | |
|---|---|---|---|---|
| Promicromonospora | 17 | 0.00574614 | 0.000574 | 0.00017964 |
| Propionicimonas | 17 | 0.07953077 | 0.00133685 | 0.0013611 |
| Rhodovibrio | 17 | 0.54599547 | 0.00091361 | 0.00296867 |
| Candidatus Contubernalis | 16 | 0.00845094 | 0.00080278 | 0.00022236 |
| Candidatus Phlomobacter | 16 | 0.01259605 | 0.00091291 | 0.00031036 |
| Candidatus Protochlamydia | 16 | 0.15456978 | 0.00123353 | 0.00165418 |
| Citrobacter | 16 | 0.20497895 | 0.00060928 | 0.00306975 |
| Ferrimonas | 16 | 0.00915378 | 0.000574 | 0.00017197 |
| Lentzea | 16 | 0.10832373 | 0.0004865 | 0.00080908 |
| Ochrobactrum | 16 | 0.09972741 | 0.00076326 | 0.0009927 |
| Pseudobutyrivibrio | 16 | 0.04814388 | 0.00072271 | 0.00065136 |
| Pyrobaculum | 16 | 0.00879914 | 0.00091291 | 0.0002111 |
| Erythromicrobium | 15 | 0.04524491 | 0.00181393 | 0.00090488 |
| Gallibacterium | 15 | 0.01425585 | 0.00076326 | 0.00034894 |
| Pimelobacter | 15 | 2.16574237 | 0.00123089 | 0.0126412 |
| Thiorhodococcus | 15 | 0.01810938 | 0.00060928 | 0.00023514 |
| Loktanella | 14 | 0.00336151 | 0.00060928 | 0.00012877 |
| Oceanisphaera | 14 | 0.00681501 | 0.00060928 | 0.00017264 |
| Snowella | 14 | 0.34469638 | 0.00099497 | 0.00290488 |
| Thermovenabulum | 14 | 0.03695328 | 0.00080278 | 0.00039827 |
| Aminiphilus | 13 | 0.07752894 | 0.0004865 | 0.00060234 |
| Deferribacter | 13 | 0.01382853 | 0.00074138 | 0.00020015 |
| Eubacterium | 13 | 0.77144154 | 0.00190636 | 0.00619123 |
| Hylemonella | 13 | 0.13638415 | 0.00091361 | 0.00096647 |
| Jannaschia | 13 | 0.01384786 | 0.00100781 | 0.00025651 |
| Magnetospirillum | 13 | 0.01265963 | 0.00072271 | 0.00018913 |
| Pedosphaera | 13 | 0.68122655 | 0.00188594 | 0.00742778 |
| Planctomyces | 13 | 0.10315866 | 0.00099497 | 0.00125791 |
| Sporichthya | 12 | 0.05554598 | 0.00099497 | 0.00052044 |
| Acidisoma | 11 | 0.34527011 | 0.00109867 | 0.00260945 |
| Crossiella | 11 | 0.01690189 | 0.00103335 | 0.00019927 |
| Phormidium | 11 | 0.02535283 | 0.00076326 | 0.00026465 |
| Phycicoccus | 11 | 0.04808226 | 0.00076326 | 0.00044357 |
| Pseudaminobacter | 11 | 0.0266896 | 0.00165109 | 0.00043352 |
| Xanthobacter | 11 | 0.0117653 | 0.00098118 | 0.00018697 |
| Blastochloris | 10 | 0.08513827 | 0.00074138 | 0.00085563 |
| Caldanaerobacter | 10 | 0.00725542 | 0.00101493 | 9.84E-05 |
| Granulicella | 10 | 0.00476474 | 0.00076326 | 0.00010037 |
| Catellatospora | 9 | 0.00337484 | 0.00103143 | 7.95E-05 |
| Longilinea | 9 | 0.09517455 | 0.00076326 | 0.00054706 |

| | | | | |
|---|---|---|---|---|
| Thiorhodospira | 9 | 0.0082021 | 0.00100781 | 0.00010821 |
| Halochromatium | 8 | 0.00491304 | 0.0010718 | 8.89E-05 |
| Pedomicrobium | 8 | 0.02923549 | 0.00141247 | 0.00022856 |
| Singulisphaera | 8 | 0.11616177 | 0.00602688 | 0.00144509 |
| Tindallia | 8 | 0.07959724 | 0.000574 | 0.00070565 |
| Methylocella | 7 | 0.00779338 | 0.00104386 | 0.00011437 |
| Pelobacter | 7 | 0.01058873 | 0.00181281 | 0.00011964 |
| Roseivirga | 7 | 0.027512 | 0.00111914 | 0.00032015 |
| Roseococcus | 7 | 0.00501546 | 0.00126488 | 7.96E-05 |
| Sphaerisporangium | 7 | 0.00436205 | 0.00080278 | 6.18E-05 |
| Ureibacillus | 7 | 0.08828931 | 0.00093628 | 0.00049789 |
| Zhouia | 7 | 0.01260525 | 0.00111914 | 0.00011166 |
| Aquimarina | 6 | 0.00327944 | 0.0010185 | 5.69E-05 |
| Balneimonas | 6 | 0.29253374 | 0.00191777 | 0.0019135 |
| Blastomonas | 6 | 0.02409897 | 0.00213707 | 0.00025135 |
| Candidatus Solibacter | 6 | 0.09116526 | 0.00449848 | 0.00082696 |
| Denitrobacter | 6 | 0.02883187 | 0.00157327 | 0.0002667 |
| Knoellia | 6 | 0.08941007 | 0.00050648 | 0.0004705 |
| Parapedobacter | 6 | 0.02375384 | 0.00219978 | 0.00018203 |
| Symploca | 6 | 0.00544178 | 0.0013297 | 7.66E-05 |
| Tepidibacter | 6 | 0.55587257 | 0.00305689 | 0.00308544 |
| Thiocystis | 6 | 0.00311735 | 0.00060928 | 4.72E-05 |
| Laceyella | 5 | 0.02376696 | 0.00142365 | 0.0002012 |
| Marivita | 5 | 0.00476474 | 0.00123353 | 6.05E-05 |
| Neorickettsia | 5 | 0.00439957 | 0.00099497 | 4.79E-05 |
| Frigoribacterium | 4 | 0.02620608 | 0.00336151 | 0.00028748 |
| Halothiobacillus | 4 | 0.00800747 | 0.00111914 | 6.32E-05 |
| Kytococcus | 4 | 0.08435781 | 0.00867253 | 0.00061134 |
| Rubrobacter | 4 | 0.03909508 | 0.00169039 | 0.00028234 |
| Citromicrobium | 3 | 0.00362786 | 0.0004865 | 3.62E-05 |
| Desulfitobacterium | 3 | 0.00292869 | 0.00099497 | 2.41E-05 |
| Gluconacetobacter | 3 | 0.02102902 | 0.00168478 | 0.00015092 |
| Kouleothrix | 3 | 0.01711303 | 0.00252105 | 0.00012203 |
| Novispirillum | 3 | 0.01631528 | 0.00112477 | 9.46E-05 |
| Salmonella | 3 | 0.02069536 | 0.00103335 | 0.00011429 |
| Leucothrix | 2 | 0.00501454 | 0.00266916 | 3.64E-05 |

Supplemental Table 3: Taxa identified in updated analysis with DADA2 but not with original analysis

| Genus | Count | Maximum Relative Abundance | Minimum Relative Abundance | Mean Relative Abundance |
|---|---|---|---|---|
| Cutibacterium | 178 | 58.8692498 | 0.01118443 | 3.94134097 |
| Escherichia/Shigella | 85 | 99.8775177 | 0.01175254 | 4.91545093 |
| Lawsonella | 80 | 3.04458137 | 0.00687876 | 0.18226868 |
| Ezakiella | 72 | 13.1103903 | 0.003139 | 0.22165684 |
| Methylophilus | 67 | 4.84244259 | 0.00356837 | 0.21395235 |
| Actinotignum | 53 | 64.7708383 | 0.00987882 | 0.62764323 |
| Massilia | 49 | 2.09878374 | 0.00966806 | 0.07357515 |
| Pseudarcicella | 49 | 6.17954499 | 0.00260461 | 0.17767683 |
| Howardella | 48 | 1.27793707 | 0.00737645 | 0.05258105 |
| Clostridium_sensu_stricto | 44 | 88.5600496 | 0.00498915 | 0.52225445 |
| Fastidiosipila | 43 | 6.23677331 | 0.0090704 | 0.14462697 |
| Tibeticola | 42 | 0.96564078 | 0.0045352 | 0.06188054 |
| Undibacterium | 42 | 4.58326931 | 0.00161692 | 0.17401873 |
| Agathobacter | 41 | 4.50250505 | 0.0102042 | 0.1382538 |
| Lachnoclostridium | 35 | 3.80372682 | 0.0090704 | 0.07911777 |
| Rhodoluna | 32 | 2.71432809 | 0.02415973 | 0.08979255 |
| DNF00809 | 31 | 4.14932305 | 0.00422012 | 0.14934225 |
| Acetobacteroides | 29 | 2.90714921 | 0.02319334 | 0.08408583 |
| Paludibacter | 29 | 4.16855975 | 0.00807298 | 0.08274717 |
| Tepidicella | 28 | 0.1382883 | 0.00401999 | 0.0038976 |
| Fusicatenibacter | 25 | 3.09835633 | 0.02164971 | 0.06159392 |
| Ruminococcaceae_UCG-002 | 25 | 3.14949202 | 0.00541243 | 0.05656913 |
| Lachnospiraceae_NK4A136_group | 24 | 1.39332366 | 0.01746202 | 0.03758849 |
| Alistipes | 23 | 1.10956282 | 0.00541243 | 0.02808936 |
| Propionimicrobium | 23 | 1.63002768 | 0.01284951 | 0.03154161 |
| Rikenellaceae_RC9_gut_group | 23 | 0.76923077 | 0.00405932 | 0.01624538 |
| Ruminiclostridium | 23 | 0.86117298 | 0.00289917 | 0.02691784 |
| Subdoligranulum | 22 | 2.81567489 | 0.01533541 | 0.0485694 |
| Candidatus_Planktophila | 20 | 1.11984574 | 0.00719891 | 0.02942037 |
| Cytophaga | 20 | 1.37199644 | 0.00488763 | 0.03143454 |
| hgcI_clade | 20 | 1.48596024 | 0.00438582 | 0.03209252 |
| Parasutterella | 20 | 0.90372907 | 0.00499176 | 0.03202955 |
| Peredibacter | 20 | 0.35146444 | 0.00405932 | 0.00934754 |
| Sediminibacterium | 20 | 1.94712031 | 0.00439011 | 0.02029876 |

| | | | | |
|---|---|---|---|---|
| Gallicola | 19 | 0.56841731 | 0.00418533 | 0.00881427 |
| Parvimonas | 19 | 1.77050276 | 0.00465755 | 0.02977489 |
| Cloacibacterium | 17 | 0.87519026 | 0.00209266 | 0.01516526 |
| Lachnospiraceae_UCG-004 | 17 | 0.82728592 | 0.0090704 | 0.01641026 |
| Butyricicoccus | 16 | 0.91436865 | 0.00440509 | 0.01554916 |
| CAG-56 | 16 | 0.87082729 | 0.0059789 | 0.01655213 |
| Christensenellaceae_R-7_group | 16 | 0.81524216 | 0.01081344 | 0.01188085 |
| Chitinivorax | 15 | 0.15584872 | 0.00865614 | 0.00536832 |
| Lachnospiraceae_ND3007_group | 15 | 5.76197388 | 0.00540672 | 0.0511289 |
| Marmoricola | 15 | 1.2400082 | 0.01154823 | 0.01980378 |
| Alloprevotella | 14 | 0.3693889 | 0.01029707 | 0.01006097 |
| Altererythrobacter | 14 | 0.79401932 | 0.00232878 | 0.0107363 |
| Barnesiella | 14 | 0.89434825 | 0.01082485 | 0.01443778 |
| Erysipelotrichaceae_UCG-003 | 14 | 3.10595065 | 0.00645557 | 0.0301482 |
| Pseudoglutamicibacter | 14 | 1.32965686 | 0.01397266 | 0.01955979 |
| Acidipropionibacterium | 13 | 0.05010857 | 0.00222037 | 0.0009258 |
| Murdochiella | 13 | 0.30007299 | 0.00541243 | 0.00500741 |
| Perlucidibaca | 13 | 0.19347083 | 0.00488763 | 0.0055055 |
| Qipengyuania | 13 | 0.14242478 | 0.00644538 | 0.00405183 |
| Ruminococcaceae_UCG-005 | 13 | 0.22269395 | 0.01347294 | 0.00538214 |
| Ruminococcaceae_UCG-014 | 13 | 0.69286688 | 0.0022676 | 0.00741041 |
| Sulfuritalea | 13 | 0.32045576 | 0.00676472 | 0.01048908 |
| Jonquetella | 12 | 5.42332741 | 0.00586516 | 0.03054382 |
| Lachnospiraceae_UCG-001 | 12 | 0.75371363 | 0.02239704 | 0.01259759 |
| Romboutsia | 12 | 1.05360124 | 0.01284951 | 0.01644194 |
| Ruminococcaceae_UCG-003 | 12 | 0.76149863 | 0.00293258 | 0.00911776 |
| Soonwooa | 12 | 0.58587956 | 0.00195505 | 0.01080967 |
| Lachnospiraceae_UCG-010 | 11 | 0.42089986 | 0.00559926 | 0.0076152 |
| Mucilaginibacter | 11 | 0.65879146 | 0.0183725 | 0.00748959 |
| Pelosinus | 11 | 0.70422535 | 0.00237891 | 0.00847575 |
| Vulcaniibacterium | 11 | 0.40947125 | 0.02091394 | 0.00751325 |
| Allorhizobium-Neorhizobium-Pararhizobium-Rhizobium | 10 | 1.16389872 | 0.02059414 | 0.01060757 |
| Aurantimicrobium | 10 | 0.49219911 | 0.01449584 | 0.00978738 |
| Ferruginibacter | 10 | 0.60199909 | 0.00879774 | 0.00606851 |
| Aeromonas | 9 | 0.54301763 | 0.01284577 | 0.00792742 |
| Catenibacterium | 9 | 0.30459945 | 0.01119852 | 0.00483976 |
| Millisia | 9 | 0.05120328 | 0.00254992 | 0.00086405 |
| Nubsella | 9 | 0.41769225 | 0.00237891 | 0.0056214 |
| Proteus | 9 | 71.5686275 | 0.0122444 | 0.37338724 |

| | | | | |
|---|---|---|---|---|
| Ruminococcaceae_UCG-013 | 9 | 0.09448888 | 0.0051398 | 0.00158072 |
| W5053 | 9 | 0.58737151 | 0.00950634 | 0.0057133 |
| Adhaeribacter | 8 | 20.1971705 | 0.00676553 | 0.10801714 |
| Flavonifractor | 8 | 0.56603774 | 0.0039106 | 0.00590022 |
| Fonticella | 8 | 1.09018096 | 0.01096455 | 0.01221852 |
| Oscillibacter | 8 | 0.16959523 | 0.00676472 | 0.00256598 |
| SH-PL14 | 8 | 0.25877316 | 0.00852172 | 0.00219999 |
| Tessaracoccus | 8 | 0.81781009 | 0.00540672 | 0.00769272 |
| Tyzzerella | 8 | 0.34518435 | 0.01642861 | 0.00420631 |
| Anaeroglobus | 7 | 0.91598862 | 0.00554032 | 0.0126566 |
| Azorhizophilus | 7 | 0.009997 | 0.00114857 | 0.00024729 |
| Bryobacter | 7 | 0.45479534 | 0.01398504 | 0.00438239 |
| Lachnospiraceae_UCG-003 | 7 | 0.3724605 | 0.0147394 | 0.00562885 |
| Ruminococcaceae_UCG-004 | 7 | 0.28806282 | 0.01119852 | 0.00302379 |
| Simplicispira | 7 | 0.73785612 | 0.02566711 | 0.01106673 |
| Sphingorhabdus | 7 | 0.98380816 | 0.04432379 | 0.01115093 |
| Aquincola | 6 | 0.28451883 | 0.00749663 | 0.00272051 |
| Candidatus_Methylopumilus | 6 | 0.95668941 | 0.00684269 | 0.00807858 |
| Clostridium_sensu_stricto0 | 6 | 0.36209265 | 0.02834499 | 0.00443211 |
| Dolosigranulum | 6 | 0.43280675 | 0.00612417 | 0.00505921 |
| Erysipelatoclostridium | 6 | 0.05663797 | 0.01757933 | 0.00111703 |
| Fodinicola | 6 | 1.29313104 | 0.01291114 | 0.01115718 |
| Gemmatirosa | 6 | 0.11355763 | 0.01029707 | 0.00163034 |
| Intestinibacter | 6 | 0.19927141 | 0.01082485 | 0.00318239 |
| Microvirga | 6 | 0.33341465 | 0.02092663 | 0.0034152 |
| Ruminococcaceae_NK4A214_group | 6 | 0.09229147 | 0.00839889 | 0.00115509 |
| S5-A14a | 6 | 0.15779508 | 0.00781555 | 0.00170555 |
| Terrimonas | 6 | 0.23724241 | 0.00322016 | 0.00263566 |
| Aeribacillus | 5 | 0.553796 | 0.07481493 | 0.00655121 |
| Alcaligenes | 5 | 0.31235576 | 0.01173032 | 0.00414242 |
| Blastocatella | 5 | 1.33224021 | 0.02519617 | 0.00761976 |
| Candidatus_Limnoluna | 5 | 0.40101042 | 0.06143535 | 0.00489985 |
| Cavicella | 5 | 0.43451118 | 0.01108086 | 0.00270078 |
| Chungangia | 5 | 0.82237887 | 0.0077097 | 0.00718829 |
| Dechlorobacter | 5 | 1.35716405 | 0.01377938 | 0.00875843 |
| Ellin6055 | 5 | 0.22056092 | 0.01347294 | 0.00195793 |
| Intestinimonas | 5 | 0.1670036 | 0.01564241 | 0.00297553 |
| Lachnoanaerobaculum | 5 | 1.24831957 | 0.00966806 | 0.00689111 |
| Lacunisphaera | 5 | 0.11457981 | 0.00668986 | 0.0009542 |
| Negativibacillus | 5 | 0.28210839 | 0.02162688 | 0.00244701 |

| | | | | |
|---|---|---|---|---|
| Pseudocitrobacter | 5 | 0.09385449 | 0.00459427 | 0.00085496 |
| Ruminococcaceae_UCG-001 | 5 | 0.19013635 | 0.0124718 | 0.00200524 |
| Ruminococcaceae_UCG-010 | 5 | 0.0601097 | 0.01399815 | 0.00101373 |
| Sellimonas | 5 | 0.71117562 | 0.00811008 | 0.00417985 |
| Terrisporobacter | 5 | 0.08623537 | 0.00439483 | 0.00090078 |
| Thermoactinomyces | 5 | 1.34598577 | 0.0129842 | 0.00748079 |
| Acidaminobacter | 4 | 0.24988642 | 0.00594728 | 0.00200082 |
| Agreia | 4 | 0.48853683 | 0.02011162 | 0.00344384 |
| Alloiococcus | 4 | 0.30055867 | 0.00391011 | 0.00233725 |
| Aquicella | 4 | 0.3008864 | 0.01878358 | 0.00258146 |
| Atopobacter | 4 | 0.02164971 | 0.00354014 | 0.00028443 |
| CAG-352 | 4 | 0.07267945 | 0.01823225 | 0.00073916 |
| Candidatus_Omnitrophus | 4 | 1.22501612 | 0.02042101 | 0.01121774 |
| Candidatus_Rhabdochlamydia | 4 | 0.12894907 | 0.0034014 | 0.00173485 |
| Chryseomicrobium | 4 | 0.43814914 | 0.04508058 | 0.00436019 |
| CL500-29_marine_group | 4 | 0.28418779 | 0.009997 | 0.00279056 |
| Cnuella | 4 | 0.37627193 | 0.00878966 | 0.00237132 |
| Family_XIII_AD3011_group | 4 | 0.06217729 | 0.00475317 | 0.00043484 |
| Holdemanella | 4 | 0.78374456 | 0.00554032 | 0.00659711 |
| Holdemania | 4 | 0.08503763 | 0.00405932 | 0.00065519 |
| Iamia | 4 | 0.28092496 | 0.01732235 | 0.00274419 |
| Nitrosomonas | 4 | 0.09438725 | 0.00805672 | 0.00092357 |
| Nosocomiicoccus | 4 | 0.35684356 | 0.01330887 | 0.00248185 |
| Oceanivirga | 4 | 0.089252 | 0.01098298 | 0.000723 |
| Oxalicibacterium | 4 | 0.89332449 | 0.02196547 | 0.00629207 |
| Paenarthrobacter | 4 | 0.28448561 | 0.00973538 | 0.00237907 |
| Phreatobacter | 4 | 0.12021488 | 0.01533541 | 0.00100049 |
| Rhodovastum | 4 | 1.13475177 | 0.01235824 | 0.00606313 |
| Roseateles | 4 | 0.3189138 | 0.10494874 | 0.00460041 |
| Silanimonas | 4 | 0.17803098 | 0.05926641 | 0.00214659 |
| Solobacterium | 4 | 0.21647113 | 0.0302054 | 0.00247702 |
| Stenotrophobacter | 4 | 0.51240008 | 0.07943068 | 0.00486817 |
| Sulfuricurvum | 4 | 0.10954617 | 0.01270785 | 0.00097786 |
| Turicella | 4 | 0.44177154 | 0.02165283 | 0.00446631 |
| UBA1819 | 4 | 0.26805869 | 0.00878966 | 0.00163952 |
| UC5-1-2E3 | 4 | 0.00893136 | 0.00439483 | 0.00014231 |
| WCHB1-32 | 4 | 0.80094927 | 0.02855239 | 0.00524699 |
| Xylophilus | 4 | 1.02481432 | 0.05920225 | 0.00665986 |
| Acidibacter | 3 | 0.21956575 | 0.03174639 | 0.00156735 |
| Asinibacterium | 3 | 0.45277636 | 0.0083262 | 0.0027891 |

| | | | | |
|---|---|---|---|---|
| Candidatus_Protochlamydia | 3 | 0.01756042 | 0.00586077 | 0.00017986 |
| Candidatus_Soleaferrea | 3 | 0.16236218 | 0.00751343 | 0.00130124 |
| Chroococcidiopsis_SAG023 | 3 | 0.09203952 | 0.00684269 | 0.00080507 |
| Coprobacter | 3 | 0.04293688 | 0.01172154 | 0.00041162 |
| Coxiella | 3 | 0.81578167 | 0.02853568 | 0.00456924 |
| Duganella | 3 | 1.34617409 | 0.78909613 | 0.01578414 |
| Gaiella | 3 | 0.0810441 | 0.05258386 | 0.00096933 |
| Gemmatimonas | 3 | 0.12811937 | 0.00746569 | 0.00117548 |
| JGI001001-H03 | 3 | 2.14328093 | 0.01247941 | 0.01121521 |
| Lacihabitans | 3 | 0.11356988 | 0.00475317 | 0.00066516 |
| Longimicrobium | 3 | 0.37611441 | 0.00523432 | 0.00200492 |
| Marvinbryantia | 3 | 0.03082502 | 0.00878966 | 0.0002777 |
| Nakamurella | 3 | 0.01968814 | 0.0045352 | 0.00021655 |
| Neochlamydia | 3 | 0.37917606 | 0.12494321 | 0.00423761 |
| Noviherbaspirillum | 3 | 0.12978585 | 0.02248988 | 0.00142755 |
| Opitutus | 3 | 0.05635073 | 0.0356062 | 0.00065806 |
| Ornithinimicrobium | 3 | 0.63357615 | 0.03629285 | 0.00404075 |
| Orrella | 3 | 0.01127989 | 0.00540672 | 0.00012853 |
| Pseudoflavonifractor | 3 | 0.00659225 | 0.00293673 | 7.70E-05 |
| Pseudolabrys | 3 | 0.19502229 | 0.12811937 | 0.00240928 |
| Psychroglaciecola | 3 | 0.69695489 | 0.03980892 | 0.00417191 |
| RB41 | 3 | 0.40996104 | 0.00769882 | 0.0023507 |
| Rhizobacter | 3 | 1.31233596 | 0.08377548 | 0.00775945 |
| Rhizorhapis | 3 | 0.07570841 | 0.05540473 | 0.00096631 |
| Salinispira | 3 | 0.75835212 | 0.02532768 | 0.00468493 |
| Senegalimassilia | 3 | 0.06054824 | 0.01284951 | 0.00058222 |
| Solitalea | 3 | 0.03878331 | 0.00365217 | 0.00029845 |
| Sphingoaurantiacus | 3 | 0.02905006 | 0.02498876 | 0.00041875 |
| Taeseokella | 3 | 0.64743299 | 0.09389671 | 0.00452504 |
| Aliterella_CENA595 | 2 | 0.14242478 | 0.0504862 | 0.00099439 |
| Amphiplicatus | 2 | 0.03073073 | 0.00563507 | 0.00018745 |
| Anaerobacterium | 2 | 0.05652911 | 0.04285492 | 0.00051229 |
| Anaerospora | 2 | 0.59064062 | 0.27079304 | 0.00444038 |
| Anaerovorax | 2 | 0.00760507 | 0.00391011 | 5.94E-05 |
| Angelakisella | 2 | 0.04818524 | 0.01480582 | 0.0003247 |
| Aquamicrobium | 2 | 0.56099358 | 0.02457556 | 0.0030184 |
| Arsenicitalea | 2 | 0.0158732 | 0.00662339 | 0.00011596 |
| Atopostipes | 2 | 0.05454262 | 0.02076556 | 0.00038819 |
| Bosea | 2 | 0.18779343 | 0.05635073 | 0.00125848 |
| Candidatus_Planktoluna | 2 | 0.22288262 | 0.03573677 | 0.00133309 |

| | | | | |
|---|---|---|---|---|
| Chroococcidiopsis_PCC203 | 2 | 0.02634063 | 0.00488763 | 0.00016097 |
| CM1G08 | 2 | 0.1626413 | 0.01045697 | 0.00089226 |
| Crocinitomix | 2 | 0.01990354 | 0.00876309 | 0.00014777 |
| Cuneatibacter | 2 | 0.02083686 | 0.00755173 | 0.00014633 |
| DTU089 | 2 | 0.04884601 | 0.04394832 | 0.00047832 |
| Effusibacillus | 2 | 0.05966587 | 0.0099955 | 0.00035908 |
| Eisenbergiella | 2 | 0.06346328 | 0.0549354 | 0.0006103 |
| Empedobacter | 2 | 0.07121239 | 0.00287142 | 0.00038188 |
| Eremococcus | 2 | 0.33157677 | 0.18171179 | 0.00264582 |
| Falsirhodobacter | 2 | 0.04581272 | 0.03483769 | 0.00041572 |
| Family_XIII_UCG-001 | 2 | 0.102882 | 0.0131845 | 0.00059828 |
| Flaviaesturariibacter | 2 | 0.03424977 | 0.00722922 | 0.00021381 |
| Fontimonas | 2 | 0.03512084 | 0.03193208 | 0.00034563 |
| Geminicoccus | 2 | 0.313222 | 0.0057038 | 0.00164395 |
| Globicatella | 2 | 0.04289959 | 0.02973696 | 0.00037442 |
| Glutamicibacter | 2 | 0.40276993 | 0.02817536 | 0.00222137 |
| Haematobacter | 2 | 0.0344515 | 0.01247287 | 0.00024188 |
| Herbinix | 2 | 0.32961238 | 0.10809121 | 0.0022562 |
| Immundisolibacter | 2 | 0.01464397 | 0.0085557 | 0.00011959 |
| Inhella | 2 | 0.63495951 | 0.08901549 | 0.00373183 |
| Kerstersia | 2 | 0.12429223 | 0.03951094 | 0.00084435 |
| Lachnospiraceae_FCS020_group | 2 | 0.13652809 | 0.0131845 | 0.00077171 |
| Malikia | 2 | 0.19157747 | 0.03802534 | 0.00118352 |
| Mumia | 2 | 0.02078714 | 0.01266384 | 0.00017243 |
| Neorhizobium | 2 | 0.08264773 | 0.00390691 | 0.00044616 |
| Nibribacter | 2 | 0.07551729 | 0.0280266 | 0.00053373 |
| OM60(NOR5)_clade | 2 | 0.17480335 | 0.00386556 | 0.00092097 |
| Paenisporosarcina | 2 | 0.11767149 | 0.01582546 | 0.00068813 |
| Parablastomonas | 2 | 0.00646768 | 0.00523432 | 6.03E-05 |
| Paracraurococcus | 2 | 0.03483769 | 0.01084383 | 0.00023547 |
| Parasegetibacter | 2 | 0.02817536 | 0.00782021 | 0.00018554 |
| Phaeodactylibacter | 2 | 0.06694561 | 0.03756715 | 0.00053873 |
| Proteiniphilum | 2 | 0.09758478 | 0.0059789 | 0.00053383 |
| Pseudarthrobacter | 2 | 0.49841414 | 0.09357455 | 0.00305149 |
| Pseudopropionibacterium | 2 | 0.20765557 | 0.0131845 | 0.00113835 |
| Pseudoscardovia | 2 | 0.03239508 | 0.0284495 | 0.00031363 |
| Rhodocytophaga | 2 | 0.09608597 | 0.00445991 | 0.00051828 |
| s3t2d-1089 | 2 | 0.07047926 | 0.01210947 | 0.00042572 |
| Siphonobacter | 2 | 0.01536537 | 0.01502686 | 0.00015666 |
| SN8 | 2 | 0.01730463 | 0.01045697 | 0.0001431 |

| | | | | |
|---|---|---|---|---|
| Stakelama | 2 | 0.01249438 | 0.00789422 | 0.0001051 |
| Subgroup0 | 2 | 0.26987856 | 0.250501 | 0.00268237 |
| Tannerella | 2 | 0.04695894 | 0.0114076 | 0.00030086 |
| UTCFX1 | 2 | 0.23681278 | 0.08341199 | 0.00165064 |
| Victivallis | 2 | 0.02834588 | 0.00839889 | 0.00018941 |
| 1174-901-12 | 1 | 0.07423538 | 0.07423538 | 0.00038266 |
| Acaricomes | 1 | 0.01823225 | 0.01823225 | 9.40E-05 |
| Acetitomaculum | 1 | 0.02216189 | 0.02216189 | 0.00011424 |
| Aestuariimicrobium | 1 | 0.05243987 | 0.05243987 | 0.00027031 |
| Agaricicola | 1 | 0.18361973 | 0.18361973 | 0.00094649 |
| Aliidiomarina | 1 | 0.42719168 | 0.42719168 | 0.00220202 |
| Allisonella | 1 | 0.00586516 | 0.00586516 | 3.02E-05 |
| Allofustis | 1 | 0.01081344 | 0.01081344 | 5.57E-05 |
| alphaI_cluster | 1 | 0.06296479 | 0.06296479 | 0.00032456 |
| Alsobacter | 1 | 0.03498426 | 0.03498426 | 0.00018033 |
| Alysiella | 1 | 0.02344147 | 0.02344147 | 0.00012083 |
| Aminobacter | 1 | 0.07533524 | 0.07533524 | 0.00038833 |
| Ammoniphilus | 1 | 0.76279157 | 0.76279157 | 0.00393192 |
| Amnibacterium | 1 | 0.06574252 | 0.06574252 | 0.00033888 |
| Anaerocolumna | 1 | 0.08276738 | 0.08276738 | 0.00042664 |
| Anaerosphaera | 1 | 0.00998353 | 0.00998353 | 5.15E-05 |
| Anaerotruncus | 1 | 0.05209215 | 0.05209215 | 0.00026852 |
| Anthococcus | 1 | 0.00390691 | 0.00390691 | 2.01E-05 |
| Antricoccus | 1 | 0.00662339 | 0.00662339 | 3.41E-05 |
| Aquaspirillum | 1 | 0.19155878 | 0.19155878 | 0.00098742 |
| Asanoa | 1 | 0.15974905 | 0.15974905 | 0.00082345 |
| ATCC-39006 | 1 | 0.00418533 | 0.00418533 | 2.16E-05 |
| Azotobacter | 1 | 0.25811336 | 0.25811336 | 0.00133048 |
| Basilea | 1 | 0.00174606 | 0.00174606 | 9.00E-06 |
| Bauldia | 1 | 0.11318436 | 0.11318436 | 0.00058343 |
| Belnapia | 1 | 0.02461524 | 0.02461524 | 0.00012688 |
| Bergeriella | 1 | 0.01583531 | 0.01583531 | 8.16E-05 |
| Brevifollis | 1 | 0.01647447 | 0.01647447 | 8.49E-05 |
| Brucella | 1 | 0.00749663 | 0.00749663 | 3.86E-05 |
| BSV13 | 1 | 0.27988259 | 0.27988259 | 0.00144269 |
| Burkholderia-Caballeronia-Paraburkholderia | 1 | 0.04559964 | 0.04559964 | 0.00023505 |
| C39 | 1 | 0.20933154 | 0.20933154 | 0.00107903 |
| Caldibacillus | 1 | 0.33982782 | 0.33982782 | 0.00175169 |
| Caldimonas | 1 | 0.19535653 | 0.19535653 | 0.00100699 |

| | | | | |
|---|---|---|---|---|
| Calothrix_PCC-6303 | 1 | 0.02195052 | 0.02195052 | 0.00011315 |
| Candidatus_Accumulibacter | 1 | 0.09637598 | 0.09637598 | 0.00049678 |
| Candidatus_Aquiluna | 1 | 0.1382451 | 0.1382451 | 0.0007126 |
| Candidatus_Finniella | 1 | 0.17092841 | 0.17092841 | 0.00088107 |
| Candidatus_Flaviluna | 1 | 0.01048878 | 0.01048878 | 5.41E-05 |
| Candidatus_Hamiltonella | 1 | 0.02422648 | 0.02422648 | 0.00012488 |
| Candidatus_Hepatincola | 1 | 0.01700753 | 0.01700753 | 8.77E-05 |
| Candidatus_Jidaibacter | 1 | 0.05259836 | 0.05259836 | 0.00027113 |
| Candidatus_Megaira | 1 | 0.57467635 | 0.57467635 | 0.00296225 |
| Candidatus_Methylomirabilis | 1 | 0.00563507 | 0.00563507 | 2.90E-05 |
| Candidatus_Paracaedibacter | 1 | 0.38765099 | 0.38765099 | 0.0019982 |
| Candidatus_Saccharimonas | 1 | 0.04311805 | 0.04311805 | 0.00022226 |
| Candidatus_Scalindua | 1 | 0.02414558 | 0.02414558 | 0.00012446 |
| Candidatus_Symbiobacter | 1 | 0.06225648 | 0.06225648 | 0.00032091 |
| Candidatus_Udaeobacter | 1 | 0.02721119 | 0.02721119 | 0.00014026 |
| Caproiciproducens | 1 | 0.11990008 | 0.11990008 | 0.00061804 |
| Catonella | 1 | 0.0068028 | 0.0068028 | 3.51E-05 |
| Cellulosibacter | 1 | 0.02380386 | 0.02380386 | 0.0001227 |
| Cereibacter | 1 | 0.39545788 | 0.39545788 | 0.00203844 |
| Citreitalea | 1 | 0.00523432 | 0.00523432 | 2.70E-05 |
| Clostridium_sensu_stricto4 | 1 | 0.00354014 | 0.00354014 | 1.82E-05 |
| Conchiformibius | 1 | 0.90620752 | 0.90620752 | 0.00467117 |
| Conexibacter | 1 | 0.03464318 | 0.03464318 | 0.00017857 |
| Cosenzaea | 1 | 1.83210784 | 1.83210784 | 0.00944386 |
| Craurococcus | 1 | 0.23101889 | 0.23101889 | 0.00119082 |
| Cricetibacter | 1 | 0.00260461 | 0.00260461 | 1.34E-05 |
| Deefgea | 1 | 0.15732301 | 0.15732301 | 0.00081094 |
| Defluviicoccus | 1 | 0.11055891 | 0.11055891 | 0.00056989 |
| Defluviitaleaceae_UCG-011 | 1 | 0.04248475 | 0.04248475 | 0.00021899 |
| Desulfitibacter | 1 | 0.01038278 | 0.01038278 | 5.35E-05 |
| Desulfomicrobium | 1 | 0.15364355 | 0.15364355 | 0.00079198 |
| Dielma | 1 | 0.04114619 | 0.04114619 | 0.00021209 |
| Dinghuibacter | 1 | 0.05064672 | 0.05064672 | 0.00026107 |
| Diplosphaera | 1 | 0.87511124 | 0.87511124 | 0.00451088 |
| Dolosicoccus | 1 | 0.00719891 | 0.00719891 | 3.71E-05 |
| Domibacillus | 1 | 0.06826405 | 0.06826405 | 0.00035188 |
| E1B-B3-114 | 1 | 0.24181548 | 0.24181548 | 0.00124647 |
| Enterorhabdus | 1 | 0.1148008 | 0.1148008 | 0.00059176 |
| Estrella | 1 | 0.00270621 | 0.00270621 | 1.39E-05 |
| Ethanoligenens | 1 | 0.01626413 | 0.01626413 | 8.38E-05 |

| | | | | |
|---|---|---|---|---|
| Extensimonas | 1 | 0.00633192 | 0.00633192 | 3.26E-05 |
| F0058 | 1 | 0.02868617 | 0.02868617 | 0.00014787 |
| Faecalitalea | 1 | 0.01582546 | 0.01582546 | 8.16E-05 |
| Falsibacillus | 1 | 0.00931511 | 0.00931511 | 4.80E-05 |
| Ferribacterium | 1 | 0.20649069 | 0.20649069 | 0.00106439 |
| Ferritrophicum | 1 | 0.07613972 | 0.07613972 | 0.00039247 |
| FFCH7168 | 1 | 0.03079861 | 0.03079861 | 0.00015876 |
| Filimonas | 1 | 0.06762087 | 0.06762087 | 0.00034856 |
| Galbitalea | 1 | 0.011338 | 0.011338 | 5.84E-05 |
| GCA-900066575 | 1 | 0.04441747 | 0.04441747 | 0.00022896 |
| GCA-900066755 | 1 | 0.01100877 | 0.01100877 | 5.67E-05 |
| GKS98_freshwater_group | 1 | 0.08030194 | 0.08030194 | 0.00041393 |
| Gottschalkia | 1 | 0.01638371 | 0.01638371 | 8.45E-05 |
| Gracilibacillus | 1 | 0.04027386 | 0.04027386 | 0.0002076 |
| Gracilibacter | 1 | 0.09875847 | 0.09875847 | 0.00050906 |
| Helcobacillus | 1 | 0.0395745 | 0.0395745 | 0.00020399 |
| Herbiconiux | 1 | 0.03578458 | 0.03578458 | 0.00018446 |
| Hespellia | 1 | 0.00659225 | 0.00659225 | 3.40E-05 |
| Holophaga | 1 | 0.01315746 | 0.01315746 | 6.78E-05 |
| Hungatella | 1 | 0.00760507 | 0.00760507 | 3.92E-05 |
| Hydrogenispora | 1 | 0.11687705 | 0.11687705 | 0.00060246 |
| Hydrogenoanaerobacterium | 1 | 0.02083686 | 0.02083686 | 0.00010741 |
| Hydrotalea | 1 | 0.01477043 | 0.01477043 | 7.61E-05 |
| Ignavigranum | 1 | 0.03079861 | 0.03079861 | 0.00015876 |
| Incertae_Sedis | 1 | 0.00947175 | 0.00947175 | 4.88E-05 |
| Isobaculum | 1 | 0.0122549 | 0.0122549 | 6.32E-05 |
| Jatrophihabitans | 1 | 0.065987 | 0.065987 | 0.00034014 |
| Jeotgalibacillus | 1 | 0.52858421 | 0.52858421 | 0.00272466 |
| Kandleria | 1 | 0.00429355 | 0.00429355 | 2.21E-05 |
| Komagataeibacter | 1 | 0.05443629 | 0.05443629 | 0.0002806 |
| Lachnospiraceae_FE2018_group | 1 | 0.05113993 | 0.05113993 | 0.00026361 |
| Lachnospiraceae_NC2004_group | 1 | 0.04186211 | 0.04186211 | 0.00021578 |
| Lachnospiraceae_UCG-009 | 1 | 0.10458332 | 0.10458332 | 0.00053909 |
| Lacibacter | 1 | 0.03005372 | 0.03005372 | 0.00015492 |
| Lactonifactor | 1 | 0.02197416 | 0.02197416 | 0.00011327 |
| Larkinella | 1 | 0.01719691 | 0.01719691 | 8.86E-05 |
| Lechevalieria | 1 | 0.20366225 | 0.20366225 | 0.00104981 |
| Leptolyngbya_PCC-6306 | 1 | 0.03944551 | 0.03944551 | 0.00020333 |
| Luteitalea | 1 | 0.15992803 | 0.15992803 | 0.00082437 |
| Lutibacter | 1 | 0.00439011 | 0.00439011 | 2.26E-05 |

| | | | | |
|---|---|---|---|---|
| Lutispora | 1 | 0.61895551 | 0.61895551 | 0.00319049 |
| Lysinimonas | 1 | 0.04814881 | 0.04814881 | 0.00024819 |
| Mailhella | 1 | 0.05339446 | 0.05339446 | 0.00027523 |
| Marinagarivorans | 1 | 0.00380253 | 0.00380253 | 1.96E-05 |
| Marinilutecoccus | 1 | 0.00781555 | 0.00781555 | 4.03E-05 |
| Mariniradius | 1 | 0.05340929 | 0.05340929 | 0.00027531 |
| Microcella | 1 | 0.05635073 | 0.05635073 | 0.00029047 |
| Micropruina | 1 | 0.07963897 | 0.07963897 | 0.00041051 |
| Mitsuokella | 1 | 0.01108064 | 0.01108064 | 5.71E-05 |
| MM2 | 1 | 0.04829115 | 0.04829115 | 0.00024892 |
| MN22.2a | 1 | 0.02066193 | 0.02066193 | 0.00010651 |
| MND1 | 1 | 0.12372704 | 0.12372704 | 0.00063777 |
| Muricoccus | 1 | 0.07444908 | 0.07444908 | 0.00038376 |
| Nafulsella | 1 | 0.06229667 | 0.06229667 | 0.00032112 |
| Neo-b11 | 1 | 0.00563507 | 0.00563507 | 2.90E-05 |
| Nostoc_PCC-7524 | 1 | 0.03215188 | 0.03215188 | 0.00016573 |
| Oceanobacillus | 1 | 0.01541941 | 0.01541941 | 7.95E-05 |
| Oleiphilus | 1 | 0.02380386 | 0.02380386 | 0.0001227 |
| Oligoflexus | 1 | 0.01502686 | 0.01502686 | 7.75E-05 |
| OM27_clade | 1 | 0.02379645 | 0.02379645 | 0.00012266 |
| Ottowia | 1 | 0.32659352 | 0.32659352 | 0.00168347 |
| Paeniglutamicibacter | 1 | 0.00947175 | 0.00947175 | 4.88E-05 |
| Pajaroellobacter | 1 | 0.011338 | 0.011338 | 5.84E-05 |
| Parachlamydia | 1 | 0.08901549 | 0.08901549 | 0.00045884 |
| Paraclostridium | 1 | 0.07121239 | 0.07121239 | 0.00036707 |
| Paramesorhizobium | 1 | 0.09758478 | 0.09758478 | 0.00050301 |
| Parasporobacterium | 1 | 0.00659225 | 0.00659225 | 3.40E-05 |
| Parvibacter | 1 | 0.01996705 | 0.01996705 | 0.00010292 |
| Pectobacterium | 1 | 0.08310249 | 0.08310249 | 0.00042836 |
| Peptoanaerobacter | 1 | 0.02357208 | 0.02357208 | 0.00012151 |
| Peptoclostridium | 1 | 0.02751505 | 0.02751505 | 0.00014183 |
| Phaselicystis | 1 | 0.03424977 | 0.03424977 | 0.00017655 |
| Phytohabitans | 1 | 0.0199456 | 0.0199456 | 0.00010281 |
| Pir4_lineage | 1 | 0.06078908 | 0.06078908 | 0.00031335 |
| Pirellula | 1 | 0.0045352 | 0.0045352 | 2.34E-05 |
| Piscinibacter | 1 | 0.17742016 | 0.17742016 | 0.00091454 |
| Planctopirus | 1 | 0.00586516 | 0.00586516 | 3.02E-05 |
| Plantibacter | 1 | 0.03040977 | 0.03040977 | 0.00015675 |
| Plastorhodobacter | 1 | 0.0045352 | 0.0045352 | 2.34E-05 |
| Plot4-2H12 | 1 | 0.01249438 | 0.01249438 | 6.44E-05 |

| | | | | |
|---|---|---|---|---|
| PMMR1 | 1 | 0.18833811 | 0.18833811 | 0.00097082 |
| Polymorphobacter | 1 | 0.43099943 | 0.43099943 | 0.00222165 |
| Prevotellaceae_NK3B31_group | 1 | 0.02380386 | 0.02380386 | 0.0001227 |
| Pseudanabaena_PCC-6802 | 1 | 0.24396194 | 0.24396194 | 0.00125754 |
| Pseudorhodoplanes | 1 | 0.03951528 | 0.03951528 | 0.00020369 |
| Quadrisphaera | 1 | 0.05247639 | 0.05247639 | 0.0002705 |
| RA | 1 | 0.00950634 | 0.00950634 | 4.90E-05 |
| Reyranella | 1 | 0.00939179 | 0.00939179 | 4.84E-05 |
| Rosenbergiella | 1 | 0.02144578 | 0.02144578 | 0.00011055 |
| Rugamonas | 1 | 0.01399815 | 0.01399815 | 7.22E-05 |
| S31 | 1 | 0.08628128 | 0.08628128 | 0.00044475 |
| Sandaracinus | 1 | 0.11203784 | 0.11203784 | 0.00057752 |
| Silvanigrella | 1 | 0.4964539 | 0.4964539 | 0.00255904 |
| Solibacillus | 1 | 0.02710958 | 0.02710958 | 0.00013974 |
| Sporobacterium | 1 | 0.00878966 | 0.00878966 | 4.53E-05 |
| Streptobacillus | 1 | 0.00270621 | 0.00270621 | 1.39E-05 |
| Sulfurimonas | 1 | 0.01317176 | 0.01317176 | 6.79E-05 |
| SV1-3 | 1 | 0.00751343 | 0.00751343 | 3.87E-05 |
| SWB02 | 1 | 0.17436992 | 0.17436992 | 0.00089881 |
| Synechococcus_IR11 | 1 | 0.02817536 | 0.02817536 | 0.00014523 |
| Tabrizicola | 1 | 0.01023982 | 0.01023982 | 5.28E-05 |
| Tahibacter | 1 | 0.00612417 | 0.00612417 | 3.16E-05 |
| Taibaiella | 1 | 0.00380253 | 0.00380253 | 1.96E-05 |
| Tepidiphilus | 1 | 0.18903592 | 0.18903592 | 0.00097441 |
| Tersicoccus | 1 | 0.00811008 | 0.00811008 | 4.18E-05 |
| Thermobifida | 1 | 1.45420043 | 1.45420043 | 0.00749588 |
| Thioclava | 1 | 0.03109223 | 0.03109223 | 0.00016027 |
| Truepera | 1 | 0.13389121 | 0.13389121 | 0.00069016 |
| Trueperella | 1 | 0.00540672 | 0.00540672 | 2.79E-05 |
| Tumebacillus | 1 | 0.37205859 | 0.37205859 | 0.00191783 |
| UTBCD1 | 1 | 0.02853568 | 0.02853568 | 0.00014709 |
| Virgisporangium | 1 | 0.14348286 | 0.14348286 | 0.0007396 |
| Weeksella | 1 | 0.33384337 | 0.33384337 | 0.00172084 |
| Zhizhongheella | 1 | 0.00329935 | 0.00329935 | 1.70E-05 |

## Supplemental Figure Legends

Supplemental Figure 1: Preliminary modeling results testing for associations with mixed urinary incontinence (MUI) versus control status. Models include data from DMM clustering as the microbial communities; filtering threshold of 0.0005. Fig 1A shows output from a preliminary

model incorporating microbial communities and clinical site showing associations with one site and MUI with cluster 5 also being associated with MUI; Fig 1B shows output from another preliminary model incorporating microbial communities, clinical site, and all clinical covariates with a different site demonstrating an association with MUI status. Figure 1C shows the output from the final model with microbial communities and clinical covariates (site removed). This summarized output is also displayed in Table 3 of the full text. Similar patterns were noted in all models including those with DMM clusters and filtering threshold of 0.0001, as well as those with DTMM clusters at both filtering thresholds.

Supplemental Figure 2: Genera identified among paired samples. Each panel depicts the relative abundance of one genus. On the left is the relative abundance from the V1-V2 amplicon, connected by a line to the right, which shows the relative abundance in the same sample when identified from the V4 amplicon. In each panel the black line summarizes the median abundances across all paired samples. The top 105 (based on abundance) genera are depicted.

Supplemental Figure 3: Stacked bar plots illustrating results when combining ASVs into microbial communities using DTMM clustering. For DTMM clustering, the total number of clusters are mathematically chosen during analysis. Clusters are numbered with each cluster further organized by those samples originating from women with MUI versus control. Suppl Figure 3A shows DTMM clustering results with the least restrictive filtering threshold of 0.0001, where 4 total clusters were identified. Suppl figure 3B shows DTMM clustering results with a the conservative filtering threshold of 0.0005, where 3 total clusters were identified. Regardless of the filtering threshold, microbial communities identified through DTMM clustering were not associated with MUI versus control status.

Supplemental Figure 4: Stacked bar plots illustrating results when combining ASVs from individual samples into microbial communities using DMM clustering. For DMM clustering a total of 6 clusters were chosen *a priori*. Clusters are numbered with each cluster further organized by those samples originating from women with MUI versus control. Suppl Figure 4A shows DMM clustering results with the least restrictive filtering threshold of 0.0001, where downstream statistical analyses also identified significant associations between Cluster 2 and Cluster 6 with MUI status (see Table 3 in main text). Both Clusters 2 & 6 include low abundances of *Lactobacillus* and a mixture of other genera. Suppl Figure 4B shows DMM clustering results with the conservative filtering threshold of 0.0005 that was used in the main analysis. In this approach, Cluster 5 (which contains moderate abundance of *Lactobacillus*) is associated with MUI while there was a trend towards Cluster 3 (containing high abundance *Lactobacillus*) being associated with controls (see Table 3 in main text).

Supplemental Figure 5: Stacked bar plots illustrating results from updated analysis when combining filtered ASVs (less restrictive threshold of 0.0001) from individual samples into bacterial communities using DMM clustering. For DMM clustering a total of 6 clusters were chosen *a priori*. Clusters are numbered with each cluster further organized by those samples originating from women with MUI versus control. Suppl Figure 5A shows DMM clustering

results with cluster membership that is significantly different between MUI and controls identified with asterisks(*). Suppl Figure 5B shows more detail about the relative abundances of various genera that contribute to each cluster.

**Supplemental Data - References**


1. Komesu YM, Richter HE, Carper B, Dinwiddie DL, Lukacz ES, Siddiqui NY, et al. The urinary microbiome in women with mixed urinary incontinence compared to similarly aged controls. Int Urogynecol J. 2018;29(12):1785-95.
2. Komesu YM, Richter HE, Dinwiddie DL, Siddiqui NY, Sung VW, Lukacz ES, et al. Methodology for a vaginal and urinary microbiome study in women with mixed urinary incontinence. Int Urogynecol J. 2017;28(5):711-20.
3. Sung VW, Borello-France D, Newman DK, Richter HE, Lukacz ES, Moalli P, et al. Effect of Behavioral and Pelvic Floor Muscle Therapy Combined With Surgery vs Surgery Alone on Incontinence Symptoms Among Women With Mixed Urinary Incontinence: The ESTEEM Randomized Clinical Trial. JAMA. 2019;322(11):1066-76.
