# Supplementary figures and images for: Updating Urinary Microbiome Analyses to Enhance Biologic Interpretation
Source: Front Cell Infect Microbiol. 2022 Jul 8;12:789439. doi: 10.3389/fcimb.2022.789439 (PMC9309214; doi:10.3389/fcimb.2022.789439)

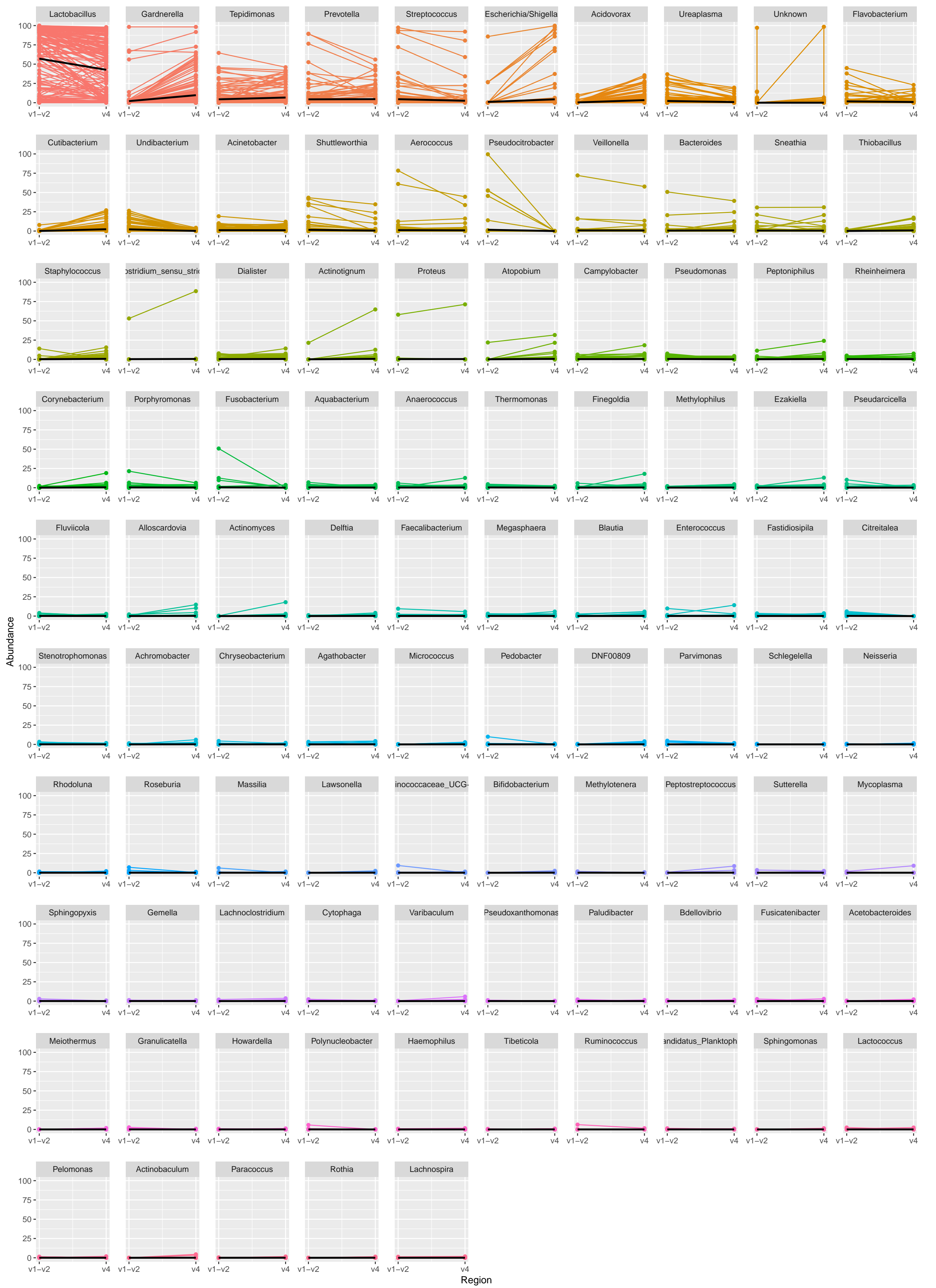

Supplement: Supplementary file 2 [file DataSheet_2.pdf]

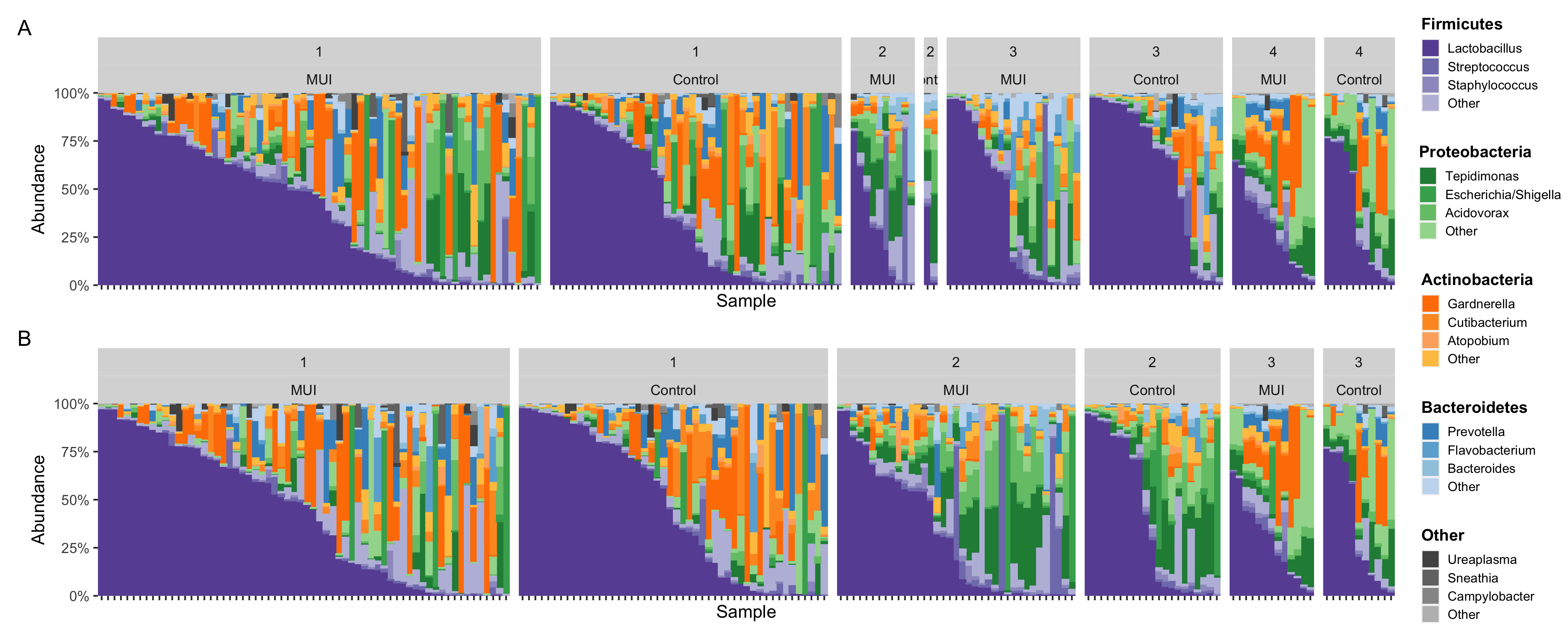

Supplement: Supplementary file 3 [file Image_1.tiff]

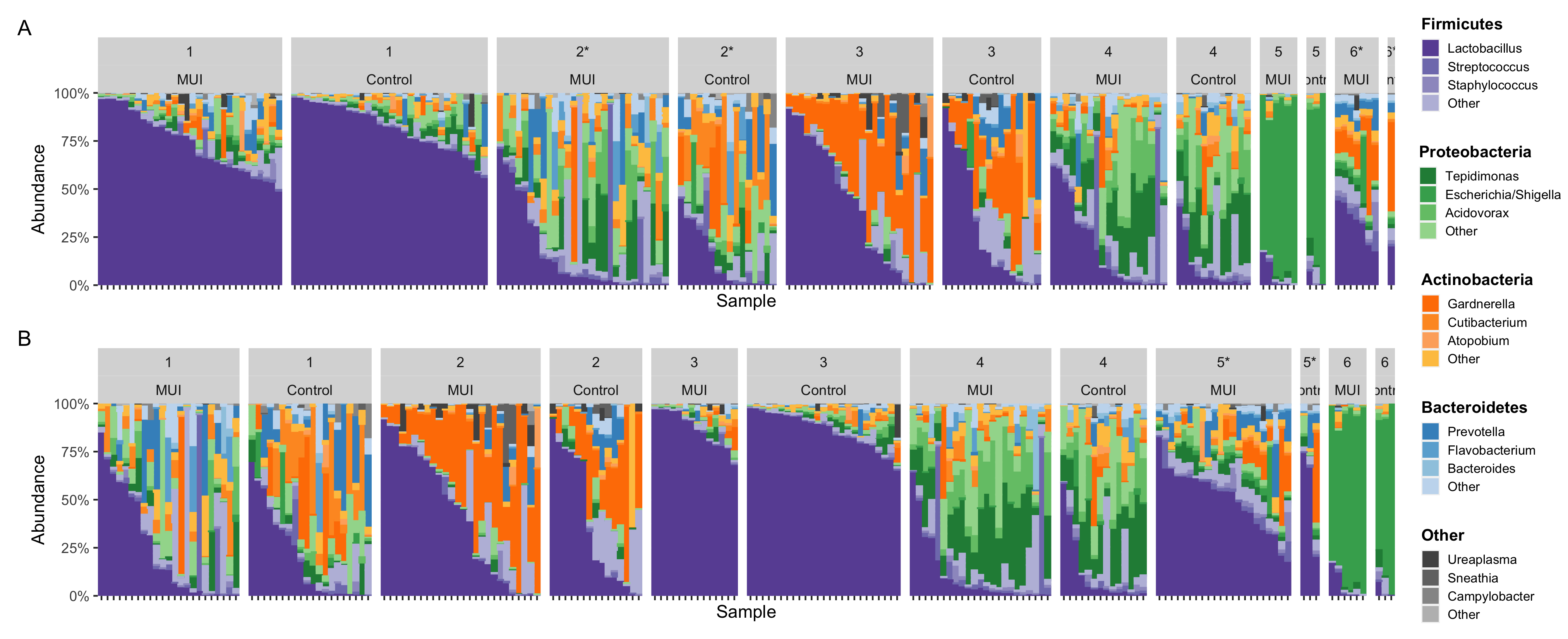

Supplement: Supplementary file 4 [file Image_2.tiff]

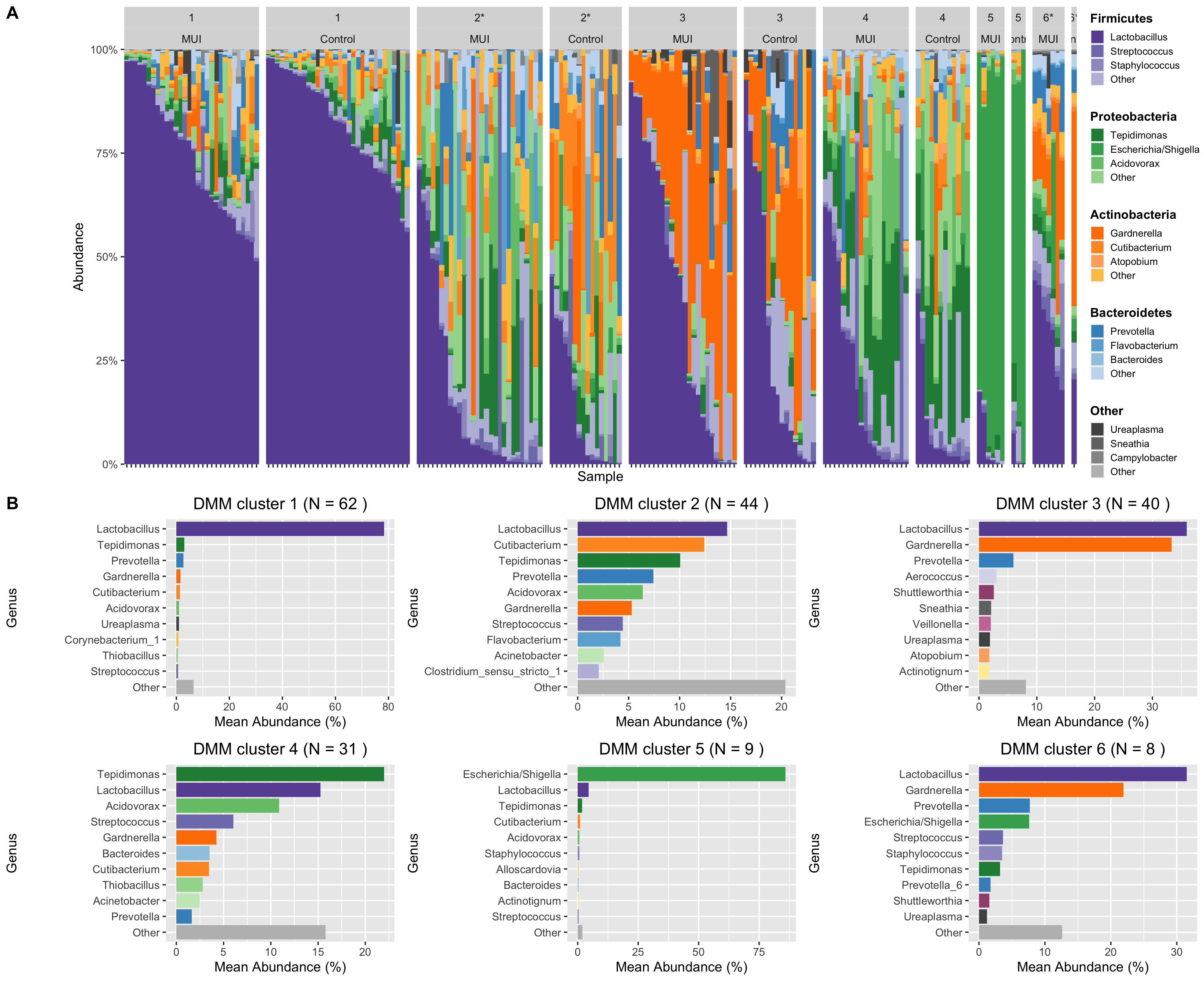

Supplement: Supplementary file 5 [file Image_3.jpeg]

## Slide 1
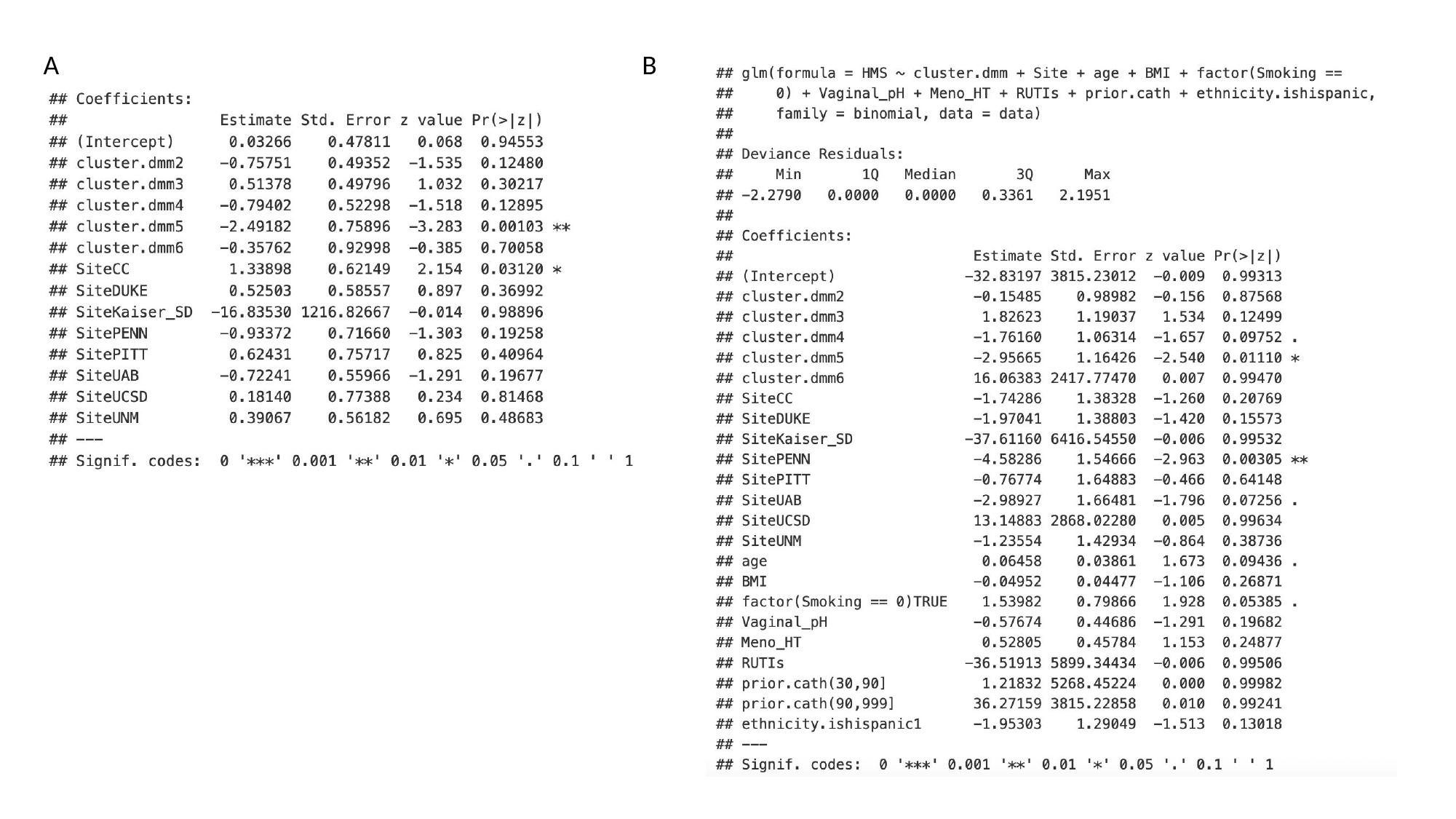

A
B

## Slide 2
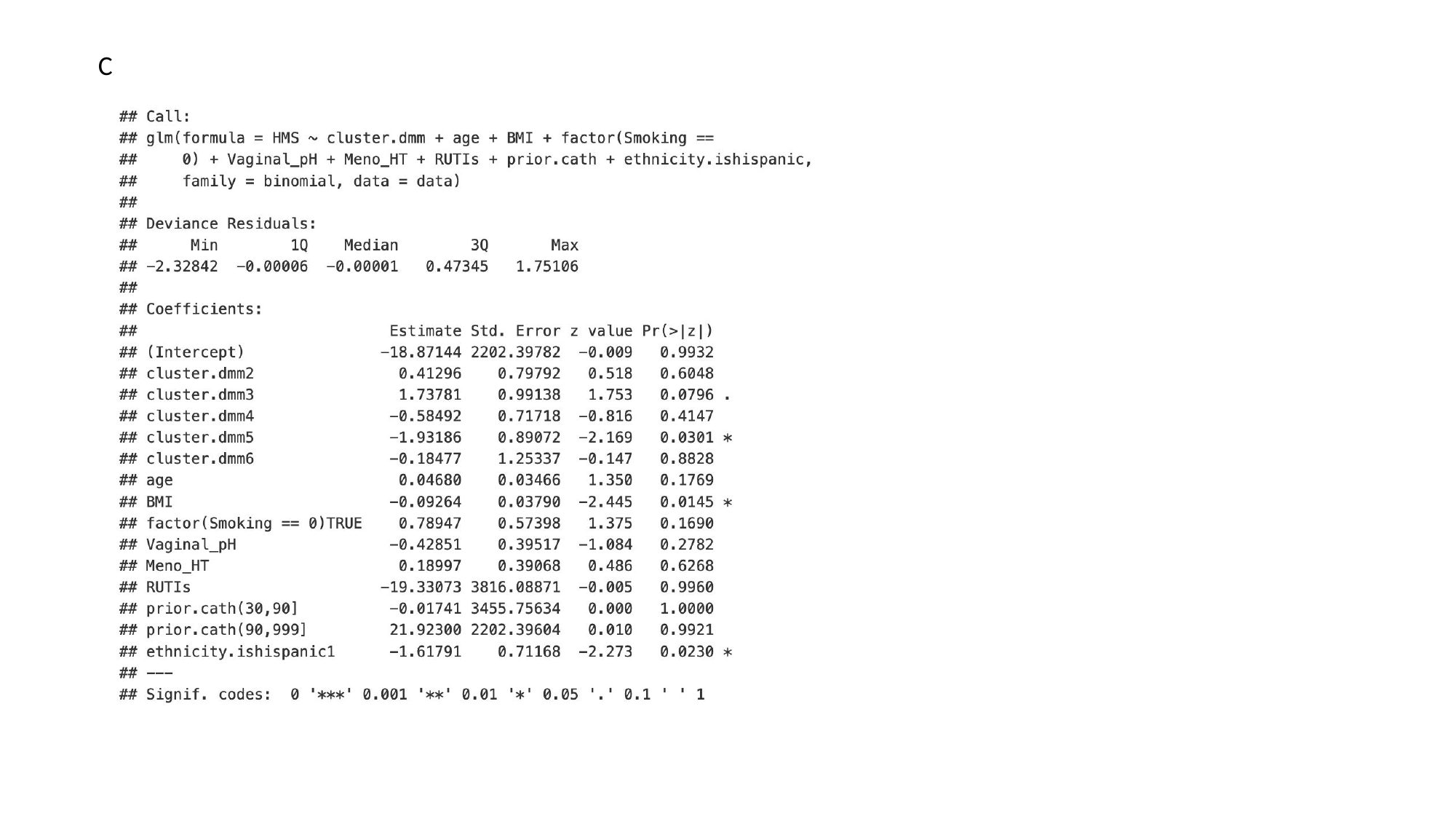

C

Supplement: Supplementary file 6 [file Presentation_1.pptx]
